# The inhibition of ABCB1/MDR1 or ABCG2/BCRP enables doxorubicin to eliminate liver cancer stem cells

**DOI:** 10.1038/s41598-021-89931-9

**Published:** 2021-05-24

**Authors:** Wang Yin, Dongxi Xiang, Tao Wang, Yumei Zhang, Cuong V. Pham, Shufeng Zhou, Guoqin Jiang, Yingchun Hou, Yimin Zhu, Yinglu Han, Liang Qiao, Phuong H.-L. Tran, Wei Duan

**Affiliations:** 1 School of Medicine, IMPACT, Institute for Innovation in Physical and Mental Health and Clinical Translation, Deakin University, Geelong, VIC 3216 Australia; 2 State Key Laboratory of Oncogenes and Related Genes, Shanghai, 200127 China; 3 Department of Biliary-Pancreatic Surgery, Renji Hospital Affiliated to Shanghai Jiao Tong University School of Medicine, Shanghai, 200127 China; 4 Shanghai Key Laboratory of Biliary Tract Disease Research, Shanghai, 200092 China; 5 Centre for Comparative Genomics, Murdoch University, Perth, WA 6150 Australia; 6 School of Nursing, Zhengzhou University, Zhengzhou, 450001 China; 7 Department of Chemical Engineering & Pharmaceutical Engineering, College of Chemical Engineering, Huaqiao University, Xiamen, 361021 China; 8 Department of General Surgery, Second Affiliated Hospital of Soochow University, 1055 Sanxiang Road, Suzhou, 215004 China; 9 Laboratory of Tumor Molecular and Cellular Biology, College of Life Sciences, Shaanxi Normal University, 620 West Chang’an Avenue, Xi’an, 710119 Shaanxi China; 10 CAS Key Laboratory of Nano-Bio Interface, Suzhou Institute of Nano-Tech and Nano-Bionics, Chinese Academy of Sciences, Suzhou, 215123 China; 11 Shanghai OneTar Biomedicine, Shanghai, 201203 China; 12 Storr Liver Centre, Westmead Institute for Medical Research, University of Sydney and Westmead Hospital, Westmead, NSW 2145 Australia

**Keywords:** Cancer stem cells, Gastrointestinal cancer

## Abstract

Two ATP-binding cassette transporters, ABCB1/MDR1 and ABCG2/BCRP, are considered the most critical determinants for chemoresistance in hepatocellular carcinoma. However, their roles in the chemoresistance in liver cancer stem cells remain elusive. Here we explored the role of inhibition of MDR1 or ABCG2 in sensitizing liver cancer stem cells to doxorubicin, the most frequently used chemotherapeutic agent in treating liver cancer. We show that the inhibition of MDR1 or ABCG2 in Huh7 and PLC/PRF/5 cells using either pharmacological inhibitors or RNAi resulted in the elevated level of intracellular concentration of doxorubicin and the accompanied increased apoptosis as determined by confocal microscopy, high-performance liquid chromatography, flow cytometry, and annexin V assay. Notably, the inhibition of MDR1 or ABCG2 led to the reversal of the chemoresistance, as evident from the enhanced death of the chemoresistant liver cancer stem cells in tumorsphere-forming assays. Thus, the elevation of effective intracellular concentration of doxorubicin via the inhibition of MDR1 or ABCG2 represents a promising future strategy that transforms doxorubicin from a traditional chemotherapy agent into a robust killer of liver cancer stem cells for patients undergoing transarterial chemoembolization.

## Introduction

Hepatocellular carcinoma (HCC) accounts for the fourth leading cause of cancer mortality with a rising incidence^1^. One of the major causes of chemotherapy failure in the treatment of HCC is multi-drug resistance^2^. Accumulating evidence has demonstrated that a small subset of liver cancer cells, termed liver cancer stem cells (LCSCs), are responsible for the initiation, propagation, maintenance, and chemoresistance of HCC^3–5^. Doxorubicin (DOX) is the most frequently used chemotherapeutic agent in the treatment of HCC^1^. However, its utility is limited by pre-existing and acquired chemoresistance^6^. Indeed, DOX used in both systemic and local therapies for HCC has limited efficacy as it kills the tumor bulk but fails to eliminate the cancer stem cell population of HCC. Thus, cancer progression inevitably follows^7^. Therefore, treatments that can effectively eliminate CSCs are urgently needed to improve the survival of patients’ with HCC. The ATP binding cassette (ABC) transporters have been identified as one of the causal factors underlying multi-drug resistance^8,9^. Among the 48 known ABC transporters, the multi-drug resistance protein 1 (MDR1, ABCB1, P-glycoprotein) and the ABC-subfamily G member 2 (ABCG2, BCRP1) are reported as the two most important determinants for the multi-drug resistance to chemotherapy in HCC^2,10^.

MDR1 is overexpressed in 80% of HCC cases^11^, and its overexpression is associated with the reduction of overall survival. Moreover, the expression of MDR1 constitutes a prognostic factor after surgical resection in patients with HCC^12^. On the other hand, ABCG2 has been regarded as the molecular determinant of the side population phenotype, which is a surrogate marker for CSC in HCC^13^. In addition, ABCG2 has been shown as an LCSC marker and is implicated in the development of chemoresistance in LCSCs^14^. Previous studies have shown that the inhibition of MDR1 or ABCG2 leads to sensitizing HCC cells to DOX^14,15^. However, many factors contribute to the resistance of LCSCs to chemotherapy, including microenvironmental stimuli, tumor dormancy, enhanced expression of ABC transporters, activation of DNA damage repair and autophagy, as well as the infection of hepatitis B virus (HBV) and hepatitis C virus (HCV)^16,17^. Whether the inhibition of the ABC transporter MDR1 or ABCG2 can reverse the resistance of LCSCs to DOX remains elusive. In this study, two HCC cell lines Huh7 and PLC/PRF/5 were used to represent HCC cells with different expression patterns of MDR1 and ABCG2, as well as the differential expression of LCSC markers (epithelial adhesion molecule (EpCAM) and CD133). In addion, these two cell lines represent different pathological backgrounds in that PLC/PRF/5 cells were derived from a patient infected with HBV whereas Huh7 cells are HBV-free. Here, we show that the inhibition of MDR1 or ABCG2 via pharmacological inhibitors or RNAi resulted in the increased intracellular concentration of DOX, which in turn enabled DOX to eradicate LCSCs and overcame chemoresistance. Thus, as long as sufficient intracellular concentration is achieved, DOX is able to eliminate LCSCs, in sharp contrast to the popular belief that chemotherapeutic agents are generally unable to eradicate CSC^18,19^.

## Results

### The expression of MDR1 and ABCG2 is elevated in the CSC population of HCC cells

The expression of ATP‐binding cassette (ABC) superfamily transporters in LCSCs has been implicated in the chemoresistance in patients with HCC^20^. However, there is a paucity of experimental evidence on the overexpression of ABC superfamily transporters in LCSCs, which can be phenotypically defined as the cells expressing both EpCAM and CD133 (EPCAM^+^–CD133^+^) in Huh7 and PLC/PRF/5 human HCC cells^21–27^.

**Figure 1:**
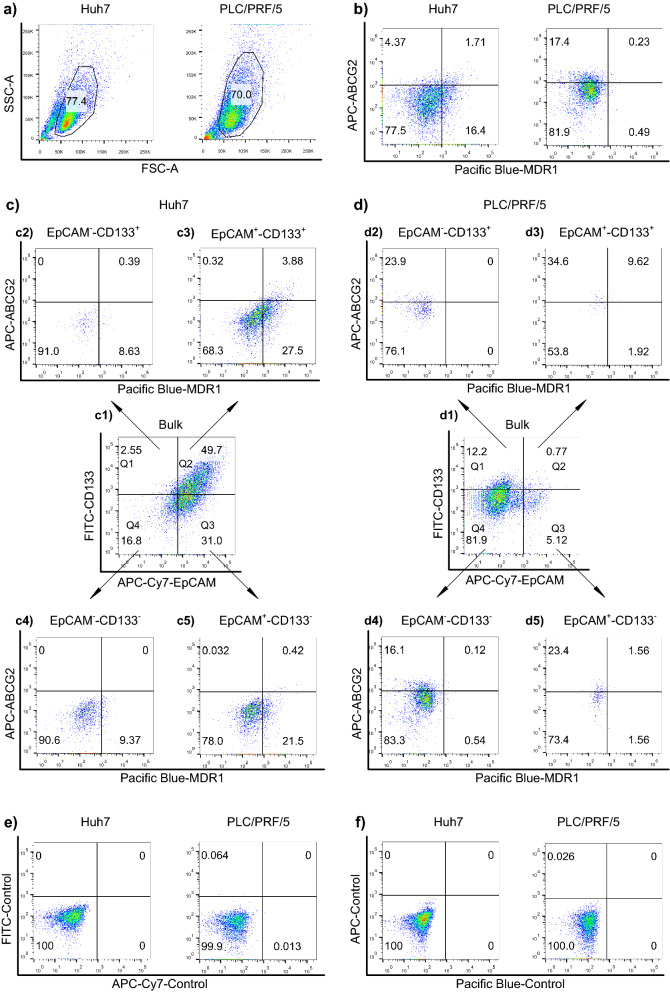
Expression of MDR1 and ABCG2 in the liver cancer stem cell (LCSC) population of Huh7 and PLC/PRF/5 cells. (**a**) The gating of viable cells using forward scatter (FSC) and side scatter (SSC). (**b**) The percentage of MDR1^+^ and ABCG2^+^ expression in the bulk tumor population. (**c1,d1**) The percentage of LCSC markers EpCAM and CD133 in the bulk tumor population. The cells shown in (**c1**) and (**d1**) were divided into four quadrants (Q1, Q2, Q3 and Q4). (**c2–c5,d2–d5**) The expression of MDR1 and ABCG2 in the cells from Q1, Q2, Q3 and Q4 of (**c1**) and (**d1**) was determined. The Huh7 and PLC/PRF/5 cells were stained with IgG isotype-matched control antibodies of (**e**) EpCAM and CD133 and (**f**) MDR1 and ABCG2. Horizontal and vertical axes denote expression intensity. One representative experiment of three is shown.

We started our flow cytometry analysis by first excluding cell debris, dead cells, and clumped cells with forward scatter (FSC) and side scatter (SSC) via a gating strategy shown in Fig. 1a. MDR1 was expressed in more than 18.1% of bulk Huh7 cells and less than 1% of the bulk PLC/PRF/5 cells (Quadrant 1 + Quadrant 4 in Fig. 1b). ABCG2 was found to express more frequently in the bulk PLC/PRF/5 cells (17.63%) than that in Huh7 cells (6.08%) (Quadrant 1 + Quadrant 2 in Fig. 1b). Next, based on immunophenotypes, LCSCs were defined as cells that are EpCAM^+^–CD133^+^ (cells in Quadrant 1 in Fig. 1c1,d1). As shown in Fig. 1, MDR1 and ABCG2 were most abundantly expressed in the EpCAM^+^–CD133^+^ subpopulation of the two cell types and least abundant in their EpCAM^–^–CD133^−^ subpopulation, which is considered as the non-CSC population (Fig. 1c2–4,d2–4). Also, the percentage of EpCAM^+^–CD133^+^ cells in Huh7 cells is much higher than that in the PLC/PRF/5 cells (49.7% and 0.77%, respectively, Fig. 1c3,d3). The specificity of the immunophenotyping was confirmed by the fact that over 99.9% of the cells exhibited negligible binding to the isotype-matched negative control antibodies (Fig. 1e,f).

### The extrusion of DOX is enhanced in EpCAM^+^–CD133^+^ LCSCs

**Figure 2:**
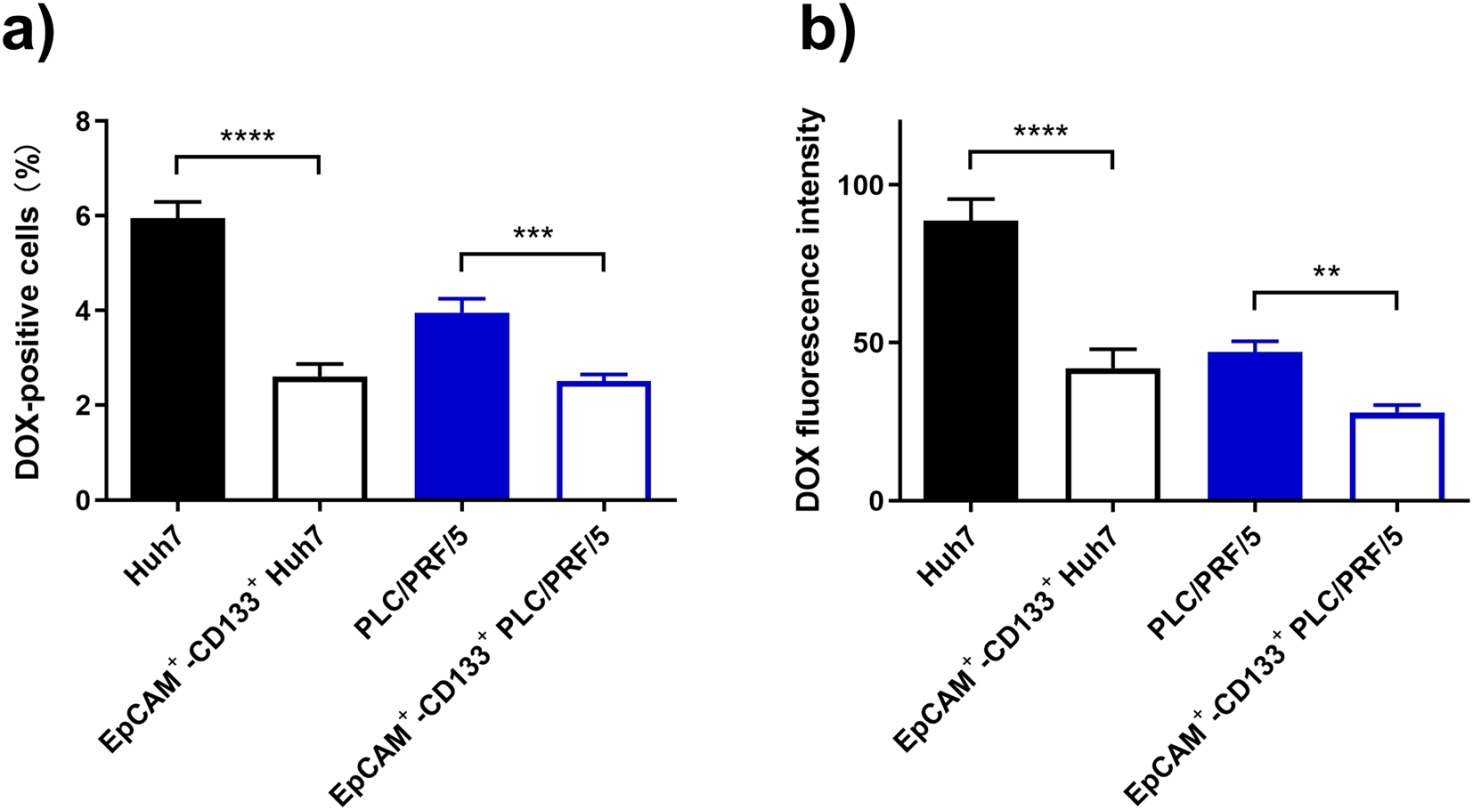
Robust extrusion of doxorubicin (DOX) by EpCAM^+^–CD133^+^ population of Huh7 and PLC/PRF/5 cells. The Huh7 or PLC/PRF/5 cells were treated with 200 nM or 100 nM of DOX for 24 h, respectively. The percentage of DOX fluorescence-positive cells (**a**), as well as the intracellular DOX fluorescence expressed as the median fluorescence intensity (**b**) in the bulk and the EpCAM^+^–CD133^+^ population of Huh7 and PLC/PRF/5 cells, were determined using flow cytometry. Data shown are means ± SD, (n = 3). ****p < 0.0001; ***p < 0.001; **p < 0.01; compared with the bulk population of Huh7 and PLC/PRF/5 cells respectively.

DOX is a substrate of both MDR1 and ABCG2^28^. Having shown the increased expression of MDR1 and ABCG2 on the EpCAM^+^–CD133^+^ subpopulation of the HCC cells and confirmed their capacity to extrude the respective substrates calcein-AM and Hoechst 33342 (Supplementary Fig. S1), we proceeded to determine if the increased expression of MDR1 and ABCG2 in the EpCAM^+^–CD133^+^ LCSCs is associated with the reduction in cellular DOX accumulation. To this end, the cellular accumulation of DOX was studied via the determination of intracellular fluorescence of DOX using flow cytometry in the bulk liver cancer cells or their CSC subpopulations. As demonstrated in Fig. 2, after treatment of DOX for 24 h, the percentage of DOX-positive cells (Fig. 2a), as well as the medium intracellular DOX fluorescence intensity (Fig. 2b), were significantly lower in the EpCAM^+^–CD133^+^ subpopulation of cells compared to that in the tumor bulk (*p* < 0.01), indicating the greater capacity of EpCAM^+^–CD133^+^ Huh7 and PLC/PRF/5 cells in extruding DOX.

### Inhibitors to MDR1 or ABCG2 are able to reverse DOX efflux in LCSCs

**Figure 3:**
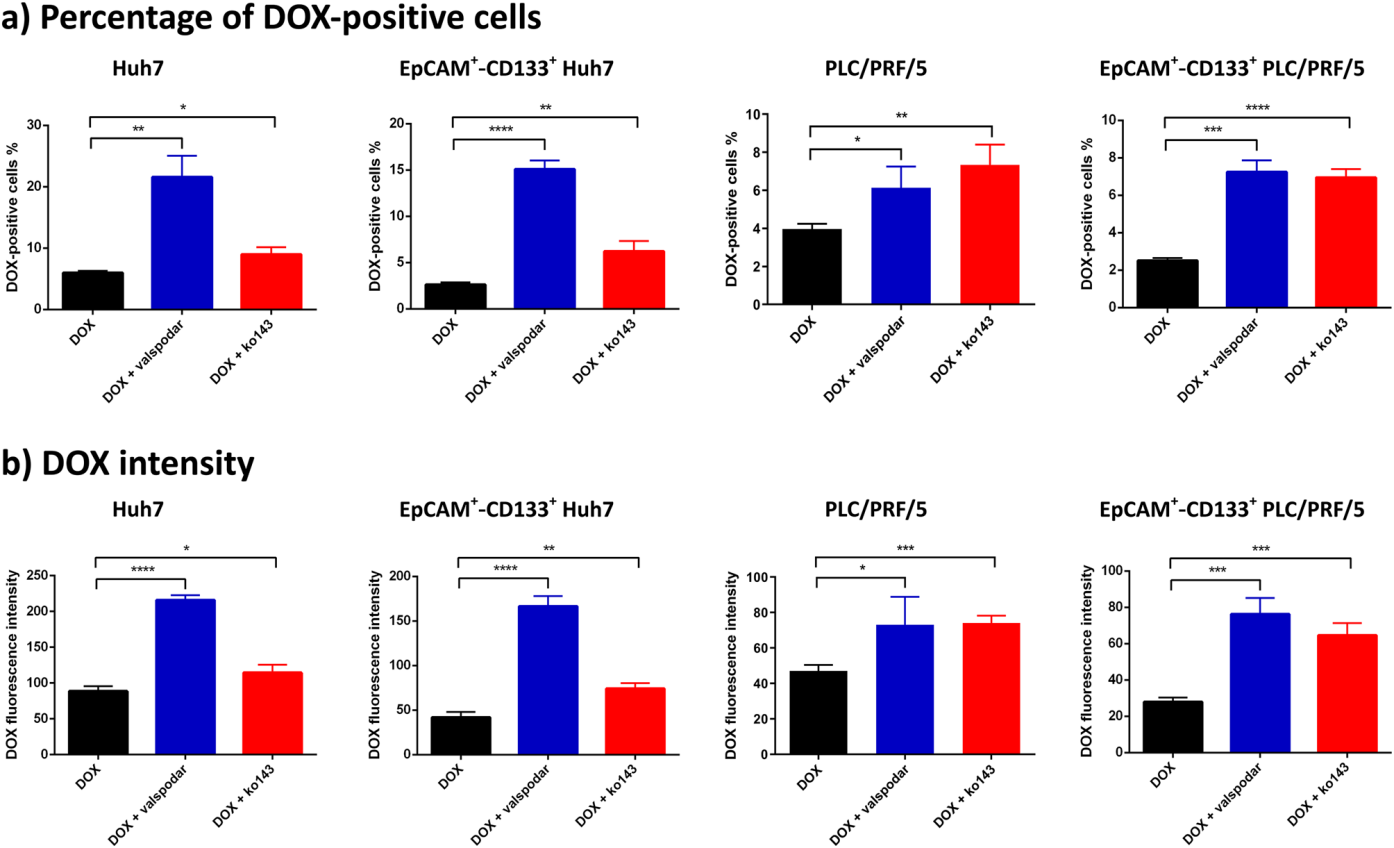
Involvement of MDR1 and ABCG2 in doxorubicin (DOX) efflux as determined by pharmacological inhibitors. The Huh7 or PLC/PRF/5 cells were treated with 200 nM or 100 nM of DOX in the presence or absence of MDR1 inhibitor valspodar (1 µM) or ABCG2 inhibitor ko143 (1 µM) for 24 h followed by flow cytometric analysis. (**a**) Percentage of DOX-positive cells; (**b**) DOX intensity followed by indicated treatments. Data shown are means ± SD, (n = 3). ****p < 0.0001; ***p < 0.001; **p < 0.01; *p < 0.05; compared with DOX treatment.

Pharmacological inhibition of drug efflux pumps including MDR1 and ABCG2 has been frequently employed in studying multi-drug resistance. Valspodar (PSC833) and ko143 are selective MDR1 and ABCG2 inhibitors, respectively^29,30^. To verify if MDR1 and ABCG2 play critical roles in DOX efflux in LCSCs, we incubated HCC cells with DOX plus valspodar or ko143 to examine if such treatments lead to increased DOX accumulation in the bulk and the EpCAM^+^–CD133^+^ HCC cells using flow cytometry. Indeed, the inhibition of MDR1 with valspodar and inhibition of ABCG2 with ko143 led to the increased percentage of DOX-positive cells (Fig. 3a) as well as elevated mean intracellular DOX fluorescence intensity (Fig. 3b) in the bulk and EpCAM^+^–CD133^+^ Huh7 and PLC/PRF/5 cells. Specifically, the treatment of valspodar in the presence of DOX resulted in a 2.62-fold and 0.74-fold increase in DOX positive cells in the bulk Huh7 and PLC/PRF/5 cells, respectively, compared to that in the DOX-only control (*p* < 0.01). Such reduction in DOX efflux was more prominent in their EpCAM^+^–CD133^+^ subpopulation as evident from a 4.77-fold (Huh7) and 1.88-fold (PLC/PRF/5) increase in the percentage of DOX-positive cells compared to that in DOX-only control (*p* < 0.001, Fig. 3a). The suppression of DOX efflux by MDR1 inhibitor was further corroborated by the increased intracellular DOX fluorescence intensity in valspodar-treated cells compared to that in DOX-only control (*p* < 0.001, Fig. 3b), demonstrating that the inhibition of MDR1 reduced DOX efflux in both the bulk and EpCAM^+^–CD133^+^ population of Huh7 and PLC/PRF/5 cells.

By the same token, the inhibition of ABCG2 using ko143 also resulted in an increased percentage of DOX-positive cells and intracellular DOX fluorescence intensity in the bulk and EpCAM^+^–CD133^+^ population of Huh7 and PLC/PRF/5 cells, respectively (*p* < 0.05, Fig. 3a,b). Notably, in Huh7 cells, the magnitude of increase in the cellular accumulation of DOX after the inhibition of ABCG2 with ko143 was not as pronounced as that treated with the MDR1 inhibitor valspodar, which may be explained, at least in part, by the approximately six-fold lower expression of ABCG2 on Huh7 cells compared to that on PLC/PRF/5 cells (Fig. 1b). The representative flow cytometric diagrams demonstrating the accumulation of DOX in the bulk and the EpCAM^+^–CD133^+^ population of Huh7 and PLC/PRF/5 cells are shown in Supplementary Fig. S2.

Next, alternative methodologies, i.e. confocal microscopy and high-performance liquid chromatography, were employed to confirm the valspodar and verapamil’s capacity in suppressing DOX efflux in the bulk HCC cells (Supplementary Figs. S3–S5). Interestingly, the fold of change in the intracellular concentration of DOX before and after the treatment of pharmacological inhibitors to MDR1 or ABCG2 was much less pronounced in the non-CSC subpopulations of cells (EpCAM^+^–CD133^−^, EpCAM^–^–CD133^+^, and EpCAM^−^–CD133^−^) in both Huh7 and PLC/PRF/5 cells compared with that in the EpCAM^+^–CD133^+^ subpopulations of these cells (Supplementary Fig. S6). Thus, we have established a causal relationship between the inhibition of MDR1 and ABCG2 and the reversal of DOX efflux via three independent yet complementary experimental approaches.

### Downregulation of MDR1 or ABCG2 via RNAi inhibits DOX efflux in LCSCs

One of the limitations inherent in the use of pharmacological inhibitors is the lack of specificity. For example, at a high dose, verapamil also inhibits ABCG2 and multi-drug resistance protein 1 (MRP1)^31,32^. Likewise, ko143 can inhibit both MDR1 and MRP1 at high concentrations (> 1 µM)^33^. Therefore, we adopted a molecular biological approach to down-regulate the expression of MDR1 and ABCG2 using RNA interference (RNAi) to verify the role of MDR1 and ABCG2 in the efflux of DOX in LCSCs.

As determined by quantitative reverse transcription PCR assay (Supplementary Fig. S7), treatment of cells with the siRNA against MDR1 for 48 h led to a ~ 78% reduction of MDR1 mRNA in both Huh7 and PLC/PRF/5 cells, while the treatment of cells with siRNA against ABCG2 led to a 71% and 81% reduction on ABCG2 mRNA in Huh7 and PLC/PRF/5 cells, respectively. There was a corresponding reduction in at least 71% of MDR1 and ABCG2 protein in these two cell lines 48 h after RNAi as determined by Western blotting (Supplementary Fig. S7). Next, cells were subject to RNAi against MDR1 or ABCG2 for 6 h followed by a further 42 h culture in the fresh culture medium to suppress the expression of MDR1 and ABCG2 followed by the incubation with DOX for a further 24 h. The intracellular DOX, along with the immunophenotyping, was quantified by flow cytometry. As expected, the downregulation of MDR1 resulted in at least a 1.52-fold increase in both the percentage of DOX-positive cells and the intracellular DOX intensity in the bulk and EpCAM^+^–CD133^+^ Huh7 cells.

**Figure 4:**
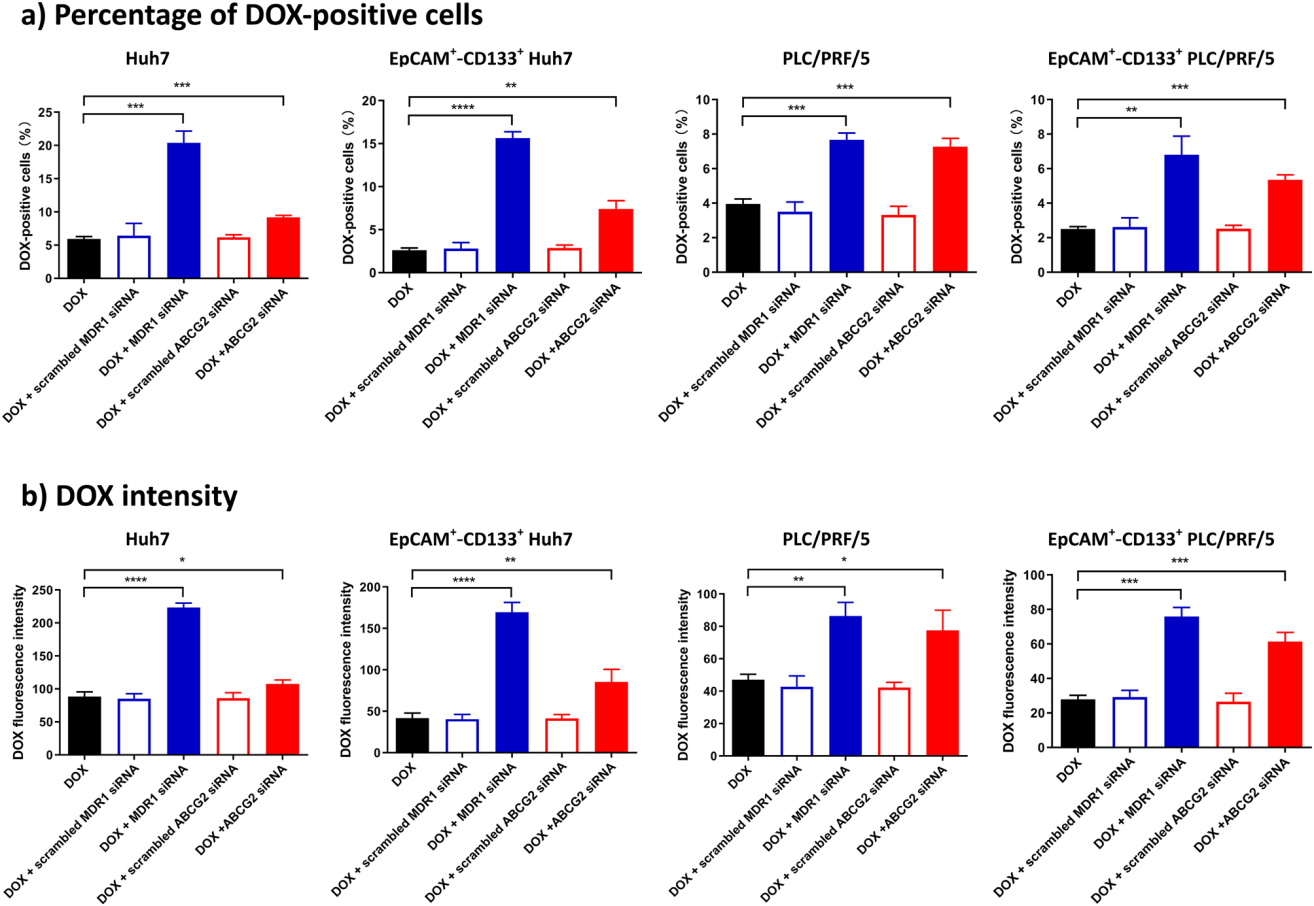
The downregulation of ABCG2 or MDR1 via RNAi and the intracellular concentration of doxorubicin (DOX). Cells were treated with siRNAs (20 nM) against MDR1 or ABCG2 for 6 h followed by a further 42 h culture in the fresh culture medium. Then the Huh7 cells or PLC/PRF/5 cells were treated with 200 nM or 100 nM of DOX for 24 h. The intracellular retention of DOX measured as (**a**) the percentage of DOX-positive cells or (**b**) their intracellular DOX fluorescence intensity was determined via flow cytometry in the bulk Huh7 and PLC/PRF/5 cells as well as in their EpCAM^+^–CD133^+^ counterparts. Data shown are means ± SD, (n = 3). ****p < 0.0001; ***p < 0.001; **p < 0.01; *p < 0.05; compared with DOX treatment.

Similarly, in the bulk and EpCAM^+^–CD133^+^ PLC/PRF/5 cells treated with siRNA against MDR1, there was also an increase (83.7%) in both the percentage of DOX-positive cells and the intracellular DOX intensity, confirming the role of MDR1 in the extrusion of DOX. As for ABCG2, its downregulation by RNAi resulted in at least a 21.4% increase in the percentage of DOX-positive cells and the intracellular DOX intensity in the bulk and EpCAM^+^–CD133^+^ Huh7 cells, as well as at least a 64.9% increase in both the percentage of DOX-positive cells and the intracellular DOX intensity in the bulk and EpCAM^+^–CD133^+^ PLC/PRF/5 cells (Fig. 4, Supplementary Fig. S8). Taken together, these data indicate an important role of MDR1 and ABCG2 in the efflux of DOX in HCC cells in general and in the LCSCs in particular.

In the context of this study, the non-CSCs can be phenotypically defined as the subpopulations of cells that are EpCAM^+^–CD133^−^, EpCAM^−^–CD133^+^ or EpCAM^–^–CD133^−^. The down-regulation of MDR1 resulted in only moderate changes in DOX efflux in these non-CSC subpopulations of both Huh7 and PLC/PRF/5 cells, while the knockdown of ABCG2 had little impact on the DOX efflux activities in these non-CSC subpopulations of Huh7 cells (Supplementary Fig. S9). The specificity of the RNAi was confirmed by the demonstration that the treatment of cells with scrambled siRNAs had little effect on the cellular accumulation of DOX (Fig. 4, Supplementary Fig. S9). Taken together, we have established that the inhibition of MDR1 or ABCG2 using either pharmacological inhibitors or RNAi resulted in a significant impairment of the DOX efflux activities in Huh7 and PLC/PRF/5 cells, especially in the EpCAM^+^–CD133^+^ subpopulation of these HCC cells.

### Inhibition of MDR1 or ABCG2 enhances DOX-induced apoptosis

**Figure 5:**
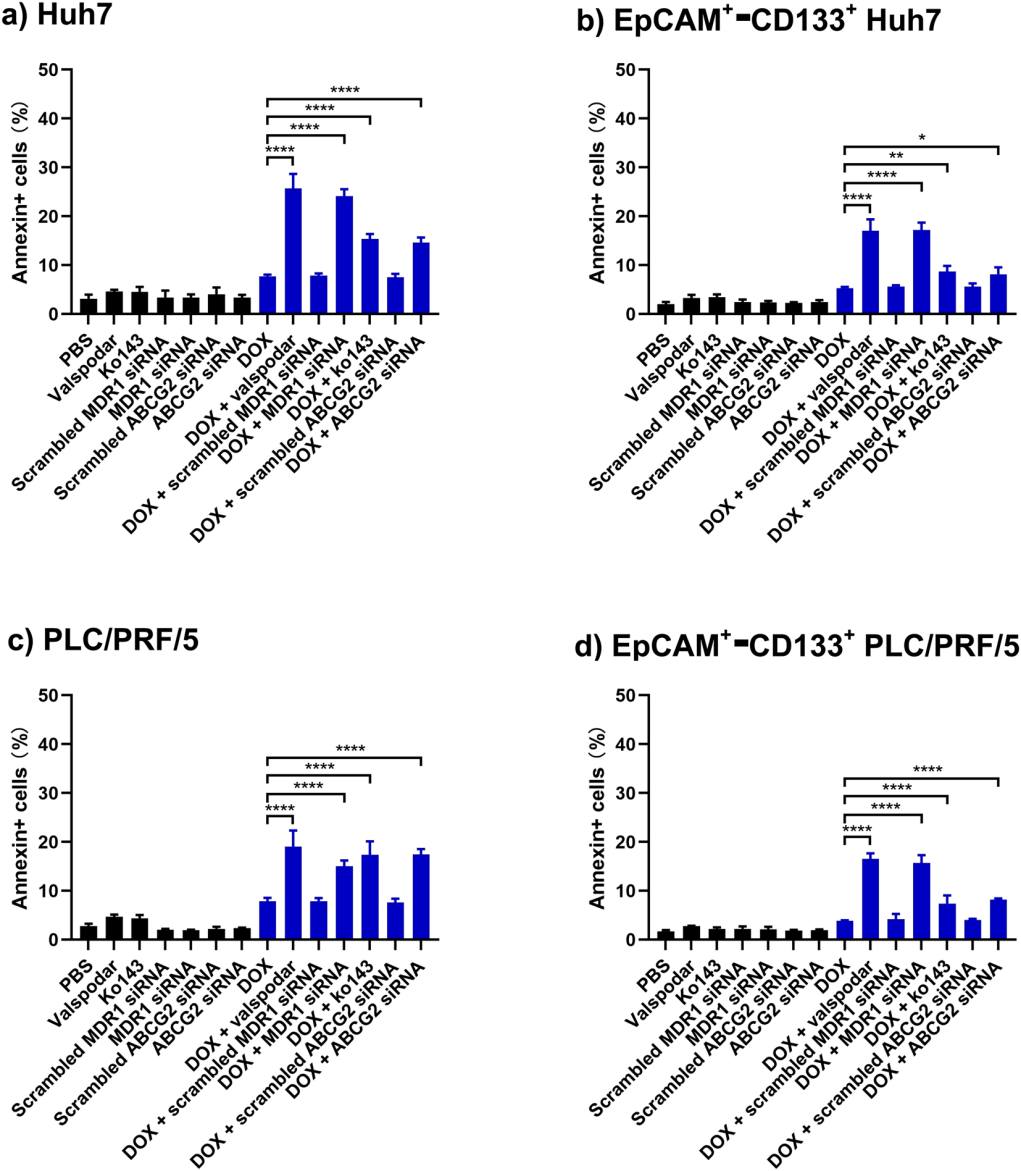
The effect of inhibition of MDR1 and ABCG2 on DOX-induced apoptosis. The cells were treated with 1 µM of MDR1 inhibitor valspodar or 1 µM of ABCG2 inhibitor ko143, and doxorubicin (DOX) (200 nM for Huh7 and 100 nM for PLC/PRF/5 cells, respectively) for 24 h. Alternatively, the cells were treated with 20 nM siRNA to MDR1 or ABCG2 for 6 h, followed by a further 42 h culture in the fresh culture medium. Then the Huh7 cells or PLC/PRF/5 cells were treated with 200 nM or 100 nM of DOX for 24 h. The total percentage of apoptotic cells as defined by the combination of 7-AAD^−^/Annexin V^+^ and 7-AAD^+^/Annexin V^+^ cells are shown for (**a**) bulk Huh7 cells, (**b**) bulk PLC/PRF/5 cells, (**c**) EpCAM^+^–CD133^+^ Hun7 cells as well as (**d**) EpCAM^+^-CD133^+^ PLC/PRF/5 cells. Data shown are mean ± SD, n = 3. ****p < 0.0001; ***p < 0.001; **p < 0.01; *p < 0.05; compared with DOX-only treatment.

DOX is known to induce apoptosis via intercalating between neighboring DNA base pairs and poisoning the topoisomerase II^34^. Having demonstrated that the inhibition of MDR1 and ABCG2 resulted in a significant elevation of intracellular levels of DOX, we proceeded to investigate the functional consequence of such inhibition in terms of induction of apoptosis in the EpCAM^+^–CD133^+^ population of HCC cells. To this end, we analyzed both the early apoptotic events via Annexin V assay and the late apoptotic events using the 7-AAD assay. The annexin V^+^ cells represent the apoptotic cells. As shown in Fig. 5, Supplementary Figs. S10 and S11, the inhibition of MDR1 with RNAi or pharmacological inhibitor valspodar combined with the treatment of DOX resulted in at least a 1.4-fold increase in the percentage of annexin V^+^ cells in both the bulk and EpCAM^+^–CD133^+^ Huh7 and PLC/PRF/5 cells compared to that in DOX-only treatment, suggesting an increase in apoptosis (*p* < 0.0001). Similarly, the combined inhibition of ABCG2 and DOX treatment resulted in up to a 1.21-fold increase in the percentage of annexin V^+^ cells in the bulk and EpCAM^+^–CD133^+^ Huh7 and PLC/PRF/5 cells (*p* < 0.001). Finally, the inhibition of either MDR1 or ABCG2 combined with DOX led to an increase in the percentage of annexin V^+^ cells in the non-CSC subpopulation of Huh7 and PLC/PRF/5 cells compared with that with DOX-only treatment (*p* < 0.05) (Supplementary Fig. S12). Collectively, the inhibition of either MDR1 or ABCG2 sensitized both the bulk and EpCAM^+^–CD133^+^ population of Huh7 and PLC/PRF/5 HCC cells to DOX-induced apoptosis. Interestingly, the ABCG2 inhibition was less effective in enhancing DOX-induced apoptosis than the MDR1 inhibition in Huh7 cells.

### Inhibition of drug efflux pump converts DOX into a CSC killer

CSCs are functionally defined by their abilities to self-renew, differentiate, and form a xenograft that resembles the parent tumor^35^. The tumorsphere formation assay is a widely used in vitro assay to analyze the self-renewal ability of CSCs^36^. The special CSCs culture condition entailed in the tumorsphere assay allows only cells with the self-renew capacity to survive and form colonies. Sphere-forming cells derived from HCC cell lines and clinical samples have been reported to possess CSC properties and are highly resistant to chemotherapeutic agents, including DOX^21,37^. Thus, we utilized this gold standard in vitro CSC assay to explore if the inhibition of drug efflux proteins in HCC cells could enable a conventional chemotherapy drug to eliminate LCSCs when its intracellular concentration is elevated.

**Figure 6:**
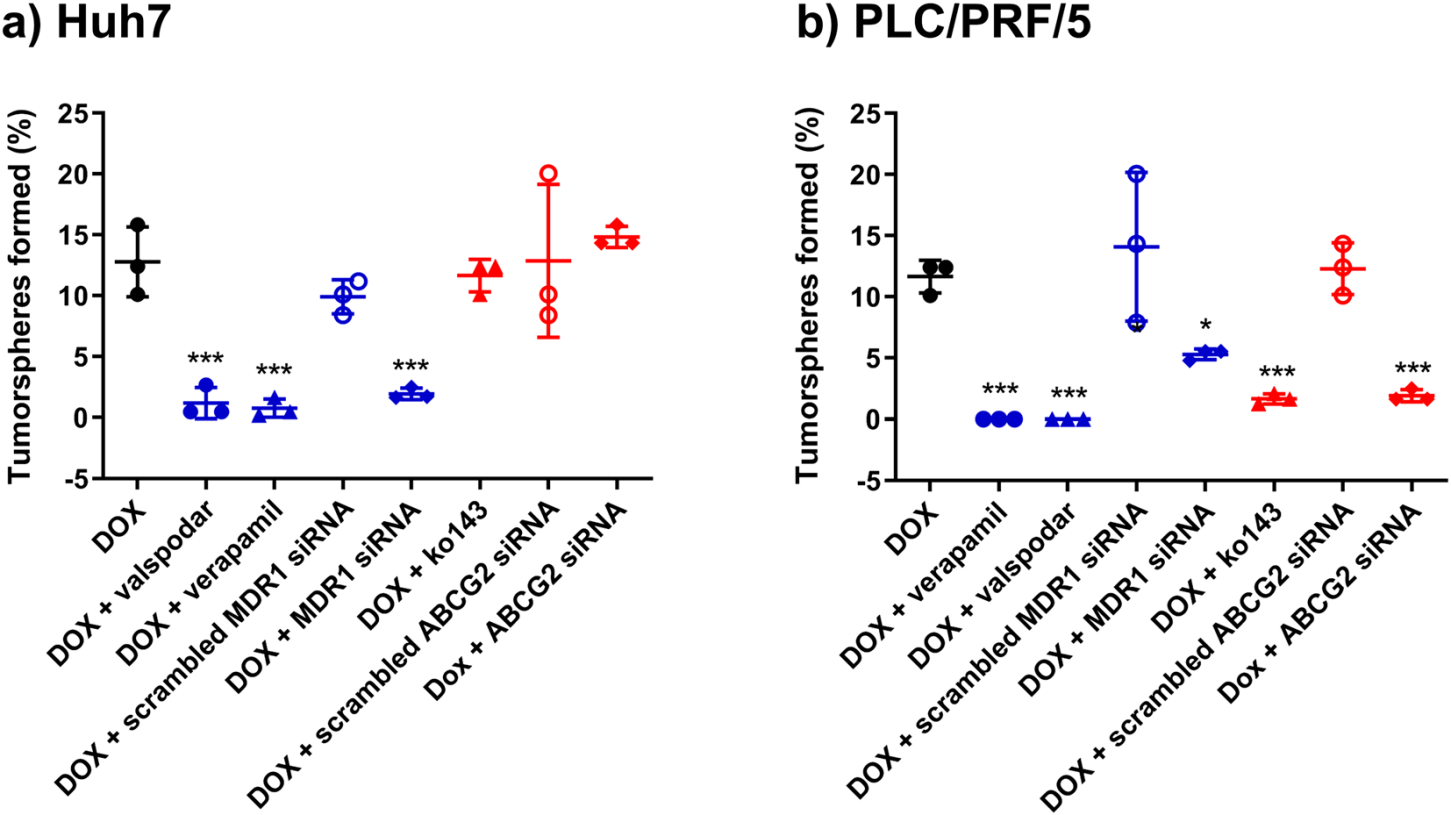
Effect of the inhibition of MDR1 and ABCG2 on the capability of doxorubicin (DOX) in the elimination of cancer stem cells in vitro. Huh7 or PLC/PRF/5 cells were treated with either doxorubicin (DOX) alone or treated with inhibitors or siRNA to MDR1 or ABCG2 first, followed by DOX treatment as described in “Materials and methods” section. Cells were then plated in ultra-low attachment plates at a density of 10, 20, or 50 cells/well and incubated for 7 days under the condition of in vitro limiting dilution assay to assess the self-renewal capacity of treated cells. The frequency of tumorsphere-forming cells for Huh7 (**a**) or PLC/PRF/5 cells (**b**) under indicated treatment is shown. Data presented are mean ± SD, n = 3. *p < 0.05; ***p < 0.001.

We began by validating the overexpression of LCSC markers EpCAM and CD133 in the Huh7 and PLC/PRF/5 sphere-forming cells. The flow cytometry analyses showed that compared with adherent cells, EpCAM expression in the sphere-forming cells in Huh7 and PLC/PRF/5 increased by 16.5% and 61.3%, respectively. In addition, CD133 expression in the sphere-forming cells in Huh7 and PLC/PRF/5 increased by 27.5% and 69.1%, respectively, demonstrating the enhanced expression of LCSC markers in the sphere-forming cells (Supplementary Fig. S13). Secondly, we validated our sphere-forming assay using salinomycin, an agent shown to kill CSCs effectively in vitro^38^. As shown in Supplementary Table ST3, the frequency of sphere-forming cells after salinomycin treatment was less than 0.3% in both Huh7 and PLC/PRF/5 cells, confirming the validity of the assay in the quantification of the stem cell frequency in vitro. Next, we treated the HCC cells with MDR1 inhibitor verapamil or valspodar and DOX for 9 days. Interestingly, such combined treatment led to a dramatic decrease in the percentage of sphere-forming cells by more than 90.7% compared to that in DOX-only treatment (*p* < 0.001) in Huh7 (Fig. 6). Complete elimination of tumorsphere-forming cells was observed in the verapamil or valspodar plus DOX treatment in PLC/PRF/5 cells (Fig. 6). To corroborate the results from pharmacological inhibitors, we used RNAi to downregulate the expression of MDR1 by approximately 70%, followed by the incubation of DOX. Such treatment resulted in an 84.8% and 54.5% decrease in the percentage of sphere-forming cells in Huh7 and PLC/PRF/5 cells, respectively, compared to that in DOX-only treatment (*p* < 0.05). As for ABCG2, its inhibition followed by the DOX treatment led to an 83% reduction of the frequency of sphere-forming cells in PLC/PRF/5 cells compared to that in DOX-only treatment (*p* < 0.001). However, a cell line-specific variation was observed in that the inhibition of ABCG2 followed by DOX treatment in Huh7 cells did not result in a statistically significant difference in the frequency of sphere-forming cells compared with that in the DOX-only control (*p* > 0.05) (Fig. 6, Supplementary Fig. S12).

Notably, the treatment of pharmacological inhibitors or siRNA against MDR1 or ABCG2 without the subsequent incubation with DOX did not lead to a decreased frequency of sphere-forming cells in either Huh7 or PLC/PRF/5 cells compared to that in the saline treatment control (*p* > 0.05) (Fig. 6, Supplementary Fig. S12). These data demonstrate that, when used alone, neither the inhibition of drug efflux proteins nor DOX was capable of killing LCSCs. Surprisingly, however, the same DOX concentration enabled the effective elimination of HCC tumor sphere-forming cells when drug efflux pumps were inhibited (Fig. 6, Supplementary Fig. S12). Thus, our results demonstrate that the inhibition of MDR1 or ABCG2 can sensitize the Huh7 and PLC/PRF/5 sphere-forming cells to DOX-induced apoptosis. Taken together, we show that, for the first time, the inhibition of drug efflux pumps in HCC cells can transform a classical chemotherapy agent regarded as incapable of eliminating CSCs into a robust LCSC killer at least in vitro.

## Materials and methods

### Cell culture

PLC/PRF/5 cell line (human HCC, ATCC CRL-8024) was purchased from ATCC. Huh7 cell line (human HCC, Japanese Collection of Research Bioresources) was kindly provided by Dr. Liang Qiao (University of Sydney, Australia). Cell line authentication was performed by CellBank Australia. The above cells were cultured in DMEM medium supplemented with 10% fetal bovine serum and 1 × Glutamax (Life Technologies, US) in a humidified atmosphere containing 5% CO_2_ at 37 °C.

### Determination of MDR1 and ABCG2 expression

Cells were dissociated into single cells, washed with PBS containing 0.1% BSA, and stained with indicated antibodies for 20 min at 4 °C. The details of the antibodies and the isotype control antibodies used in this assay are listed in Supplementary Table ST1. After thorough washing with PBS, the cells were stained with 7-AAD for 5 min, and the population of LCSCs (defined as EpCAM^+^–CD133^+^ cells) and the expression of MDR1 and ABCG2 were analyzed via flow cytometry. A minimum of 10,000 events was analyzed for each sample from three independent experiments.

### Determination of DOX accumulation

The HCC cell lines were treated with DOX (Sigma, #44583) plus verapamil (Sigma, #SML0572) or ko143 (Cayman Chemical, #15215) or siRNA against MDR1 or ABCG2 mRNA. Supplementary Table ST2 summarizes the sequences of siRNAs used in this study. The cells were then trypsinized, and the intracellular DOX accumulation was measured using flow cytometry with excitation at 488 nm and emission at 585 nm.

### Apoptosis assay

The cells were trypsinized and stained with APC/Fire 750 EpCAM antibody (1:20 dilution), VioBright FITC CD133/1 antibody (1:50 dilution), Pacific Blue Annexin V (Biolegend, #640918, 1:20 dilution), and 7-AAD (Biolegend, #420404, 1:20 dilution) for 15 min at room temperature in the dark for the determination of cell apoptosis. The apoptotic cells were quantified using flow cytometry. The corresponding isotype-matched control antibodies were used to control for background fluorescence.

### Tumorsphere formation assay

The tumorsphere assay was conducted in Corning Costar Ultra-Low attachment plates according to a previously reported protocol^39^, as described in Supplementary Materials.

### Statistical analysis

All statistical analyses were performed using GraphPad Prism 8.0 (San Diego, US). An unpaired *t*-test was used for comparisons between two experimental groups, and one-way analysis of variance (ANOVA) was used for comparisons of more than two groups. All analyses were two-tailed. Data normality was tested by Kolmogorov–Smirnov test, and parametrical statistical tests were only carried out if normality was confirmed. The homogeneity of variance was tested by Bartlett’s test. The Dunnett or the Tukey post hoc tests were conducted to compare every mean to a control mean or with every other mean only if the F value in ANOVA achieved *p* < 0.05, and there was no significant variance inhomogeneity. Otherwise, the data were converted to logarithms for further analysis. Unless stated otherwise, all results were averaged from biological triplicates, and values are reported as means ± SD. *p* < 0.05 was considered statistically significant.

## Discussion

In this study, we explored the possibility of transforming a traditional chemotherapy drug into a CSC killer via the inhibition of MDR1 and ABCG2 in HCC cells, with Huh7 and PLC/PRF/5 cells as a model. We first demonstrated overexpression of MDR1 and ABCG2 in the EpCAM^+^–CD133^+^ LCSC population. Next, we showed that the inhibition of MDR1 or ABCG2 significantly increased the cellular accumulation of DOX in both the bulk of the HCC cells and the LCSCs. The combined inhibition of MDR1 or ABCG2 and DOX treatment resulted in a substantial elevation of apoptosis cells in the LCSCs than that treated with DOX alone.

To date, a variety of LCSC surface markers have been identified, including EpCAM^40^, CD133^41^, CD90^42^, CD24^43^, CD44^44^ and CD13^45^. CD133 has been widely used to isolate LCSCs from either HCC cell lines or freshly isolated mouse HCC tissues^46,47^. CD133^+^ cells have been shown to possess classical CSC properties, including enhanced self-renewal, a broader differentiation capacity, high tumorigenicity and clonogenicity, and increased expression of stemness genes^41,48^. EpCAM^+^ HCC cells also display CSC traits, such as enhanced self-renewal, high tumorigenicity, and resistance to chemotherapy, and natural killer cell-mediated cytotoxicity^49–51^. Interestingly, although EpCAM^+^ HCC cells have been shown to initiate larger tumors compared with their EpCAM-negative counterparts in vivo, they were found to express CD133 equally. Chen et al. demonstrated that EpCAM^+^–CD133^+^ HCC cells better represent the LCSCs than the CD133^+^–EpCAM^−^, CD133^−^–EpCAM^+^ and CD133^−^–EpCAM^−^ HCC cells^23^. The combination of EpCAM^+^–CD133^+^ as LCSC markers was also successfully employed in the isolation and identification of LCSCs by a number of other laboratories^26,52,53^. Therefore, we adopted the expression of EpCAM and CD133 as a phenotypical marker to define the LCSC population in this study.

HCC is characterized by highly intratumoral and interpatient heterogeneity. The Huh7 and PLC/PRF/5 cells used in the current study represent HCC cells from different pathological backgrounds, and the cell line heterogeneity may be able to mimic, although not completely, the heterogeneity of the primary cells^54^. For example, they have different expression levels of MDR1 and ABCG2 and CSC markers, EpCAM and CD133, on their surface (Fig. 1). Furthermore, the PLC/PRF/5 cell line was derived from a patient infected with the hepatitis B virus (HBV). The HBV-encoded X antigen (HBx) is able to promote stemness and chemoresistance via the activation of PI3K/AKT signaling pathway^55–57^. In contrast, Huh7 was established from a patient without HBV infection^58,59^. Using different experimental strategies entailing pharmacological inhibitors and RNAi, we show that the inhibition of MDR1 effectively sensitized LCSCs to DOX treatment in both Huh7 and PLC/PRF/5 cells. Whereas the inhibition of ABCG2 reversed the DOX resistance in liver CSCs in PLC/PRF/5 but to a lesser extent in Huh7 (Figs. 4, 5, 6). Such discrepancies could be attributed to the following factors. First, the expression of MDR1 was higher than that of ABCG2 in both the bulk and the CSC populations of Huh7. However, the opposite was true in PLC/PRF/5 (Fig. 1). Second, in both the bulk and CSC populations of Huh7, the inhibition of MDR1 resulted in a profound increase in the intracellular concentration of DOX, while the inhibition of ABCG2 had only a moderate effect in this regard. In contrast, the inhibition of either MDR1 or ABCG2 in PLC/PRF/5 cells had a similar impact on the intracellular retaining of DOX (Figs. 3, 4). Third, the inhibition of MDR1 followed by DOX treatment induced more profound apoptosis in the bulk and CSC population of Huh7 cells than that upon ABCG2 inhibition (Fig. 5). Fourth, the inhibition of MDR1, but not the inhibition of ABCG2, conferred the DOX with the ability to eradicate the tumorsphere-forming cells effectively in Huh7. Nonetheless, the inhibition of either MDR1 or ABCG2 endowed DOX with the ability to eliminate tumorsphere-forming cells, albeit to a lesser extent in PLC/PRF/5 (Fig. 6). Taken together, our data support the hypothesis that the inhibition of MDR1 or ABCG2 enables DOX to eradicate LCSCs regardless of the HBV infection status.

The traditional chemotherapeutic agents mainly kill the rapidly dividing cancer cells. In contrast, quiescent CSCs are non-dividing and exist in the G0 phase of the cell cycle and are thus able to evade immune surveillance, resist cytotoxic insults, and repopulate the tumor after chemotherapy^18^. Indeed, LCSCs are resistant to chemotherapy, making HCC notoriously refractory to most treatment regimens^7,21,37^. Chemotherapy has been extensively explored in HCC over the past decades but has achieved minimum gain in the improvement of the overall survival in patients with HCC so far^19^. Via transarterial chemoembolization (TACE), the concentration of chemotherapeutic agents within tumors can be 10 to 100 times higher than that after systemic administration^60^. Randomized trials have suggested an increase in mean survival of 5.8 months in patients treated with TACE compared with those underwent systemic chemotherapy^61^, indicating the effectiveness of chemotherapeutic drug used for HCC treatment. However, evidence regarding the superiority of TACE over transarterial embolization (TAE) without using any chemotherapeutic agents is still lacking^62^. Our data have shown that LCSCs can be eliminated by DOX as long as sufficient intracellular accumulation of DOX can be achieved in LCSCs, thus justifying the use of DOX for the treatment of HCC.

To assess the clinical potential of eradicating LCSCs via increasing intracellular concentration of DOX, we judiciously used DOX concentration approximately five-fold lower than the 50% inhibiting concentration (IC_50_) reported for the cell line in question. Specifically, we used 200 nM DOX for Huh7 instead of the reported IC_50_ of DOX in Huh7 of 1 μM^63^, and 100 nM DOX for PLC/PRF/5 instead of the reported IC_50_ of 430 nM^64^. Under such a low DOX concentration, we demonstrated that DOX alone could not eliminate tumorsphere-forming cells in both HCC cell lines studied. However, after the inhibition of MDR1 with valspodar or verapamil, the same low dose of DOX was capable of eradicating the LCSCs as evident from the dramatical drop of the percentage of sphere-forming cells from 12.8% to at least 1.19% in Huh7 cells and from 11.65% to zero in PLC/PRF/5 cells. The fact that increased intracellular concentration of DOX upon treatment of pharmacological inhibitors to drug efflux proteins led to the elimination of tumorsphere-forming cells was corroborated by an alternative experimental approach of using RNAi to knockdown MDR1 (Figs. 4, 6, Supplementary Table ST3). Similarly, the RNAi-mediated knockdown of ABCG2 in PLC/PRF/5 cells led to the transformation of the “incompetent” DOX into a robust tumorsphere-forming cell killer (Fig. 6, Supplementary Table ST3).

Treatment with DOX alone or DOX plus inhibitors of drug efflux pumps resulted in different extent in the induction of apoptosis in Huh7 and PLC/PRF/5 cells (Fig. 5). Such a difference could be attributed to the distinct expression pattern of MDR1 and ABCG2 and the different abundance of the EpCAM^+^–CD133^+^ subpopulation in these two cell lines. In addition, PLC/PRF/5 cells were found to possess more substantial self-renewal capacity than Huh7 cells as revealed by the in vitro tumorsphere assay, indicating an enhanced stemness of PLC/PRF/5 cells and thus elevated resistance to apoptosis^65^. Finally, compared with Huh7 cells, PLC/PRF/5 cells exhibited stronger autophagic activity, which is known to contribute to the resistance to apoptosis^66^.

Apart from MDR1 and ABCG2, a few other ABC transporters, including ABCC1 (human ATP-binding cassette, subfamily C, member 1) and ABCB5 (human ATP-binding cassette, subfamily B, member 5), are also involved in the resistance to DOX^67,68^. The expression of ABCC1 in HCC is linked to a more aggressive tumor phenotype and reflects a progenitor cell origin^69^. Being highly expressed in LCSCs, ABCB5 is associated with tumor progression, chemoresistance, and recurrence in patients with HCC^70^. Furthermore, the activation of autophagy represents another mechanism underlying resistance to DOX treatment in HCC. DOX has been reported to trigger autophagy, which plays protective roles in HCC cells to the cytotoxicity of chemotherapy^71^. Therefore, the inhibition of these ABC transporter molecules and/or autophagy may also augment the anti-cancer effect of DOX in patients with HCC.

Among all the ABC transporters, MDR1 and its inhibitors have been most extensively studied. Although ample evidence suggests a rationale for using MDR1 inhibitors plus chemotherapy in cancer treatment, none of them have proven to provide clinical benefit in patients^72^. Moreover, only a few studies on the efficacy of using ABCG2 inhibitors to sensitize chemotherapy have been evaluated in clinical trials, but none of them have achieved positive clinical outcomes so far^73^. The common reasons underlying the failure of these inhibitors include safety, in vivo efficacy, and their impact on the metabolism of the chemotherapy drugs. For example, inhibitors such as valspodar and zosuquidar change the pharmacokinetics of the DOX by inhibiting the liver cytochrome P450 enzyme, leading to an increase in the systemic exposure of DOX^74,75^. Since TACE is a local treatment of liver cancer, the administration of MDR1 and ABCG2 inhibitors via TACE is expected to enhance the efficacy of DOX to kill HCC while minimally affect the systemic exposure of DOX.

Recent studies demonstrated that HCC cells surviving DOX treatment exhibit increased migratory potential and decreased susceptibility to further chemotherapeutic treatment, a sign of enrichment of CSC in the total tumor cell populations^76^. A prevailing view embraced by many cancer researchers holds that DOX is intrinsically incapable of eliminating CSCs in solid cancers^77–79^. On the contrary, our results suggest that the efficacy of DOX in cancer treatment has been underappreciated by the medical fraternity. We show here that as long as one can raise the intracellular DOX concentration, DOX may function as an effective CSC eliminator (Supplementary Fig. S4, Fig. 6).

In conclusion, our study shows that DOX can function as an effective LCSC eradicator as long as sufficient intracellular concentration is achieved in LCSCs. In the clinic, a large portion of HCC patients is diagnosed with intermediate HCC and are treated with TACE. Given none of the first-line or second-line agents used in systemic therapies are capable of eradicating LCSCs, our findings suggest that a promising future direction to improve the survival of patients HCC is to develop smart drug delivery systems for TACE in which the intracellular concentration of DOX in HCC cells is elevated via a combination of pharmacological inhibitors, RNAi, antisense oligonucleotides and/or gene editing.

## Supplementary Information

Supplementary Information.

## Abbreviations

HCC: Hepatocellular carcinoma
LCSC: Liver cancer stem cells
CSC: Cancer stem cells
DOX: Doxorubicin
ABC transporter: ATP‐binding cassette transporter
ABCB1/MDR1: ATP-binding cassette sub-family B member 1/multidrug resistance protein 1
ABCG2/BCRP: ATP-binding cassette sub-family G member 2/breast cancer resistance protein
FSC: Forward scatter
SSC: Side scatter
EpCAM: Epithelial cell adhesion molecule

## References

[1] Villanueva A (2019). Hepatocellular carcinoma. N. Engl. J. Med..

[2] Ceballos MP, Rigalli JP, Ceré LI, Semeniuk M, Catania VA, Ruiz ML, Transporters ABC (2019). Regulation and association with multidrug resistance in hepatocellular carcinoma and colorectal carcinoma. Curr. Med. Chem..

[3] Clarke MF (2019). Clinical and therapeutic implications of cancer. Stem Cells.

[4] Lytle NK, Barber AG, Reya T (2018). Stem cell fate in cancer growth, progression and therapy resistance. Nat. Rev. Cancer.

[5] Saygin C, Matei D, Majeti R, Reizes O, Lathia JD (2019). Targeting cancer stemness in the clinic: From hype to hope. Cell Stem Cell.

[6] Cox J, Weinman S (2016). Mechanisms of doxorubicin resistance in hepatocellular carcinoma. Hepatic Oncol..

[7] Vu NB, Nguyen TT, Tran LC-D, Do CD, Nguyen BH, Phan NK, Pham PV (2013). Doxorubicin and 5-fluorouracil resistant hepatic cancer cells demonstrate stem-like properties. Cytotechnology.

[8] Liu Z, Delavan B, Roberts R, Tong W (2017). Lessons learned from two decades of anticancer drugs. Trends Pharmacol. Sci..

[9] Pasello M, Giudice AM, Scotlandi K (2020). The ABC subfamily A transporters: Multifaceted players with incipient potentialities in cancer. Semin. Cancer Biol..

[10] Sun Z, Zhao Z, Li G, Dong S, Huang Z, Ye L, Liang H, Qu J, Ai X, Zhang W, Chen X (2010). Relevance of two genes in the multidrug resistance of hepatocellular carcinoma: in vivo and clinical studies. Tumori.

[11] Ng IO, Liu CL, Fan ST, Ng M (2000). Expression of P-glycoprotein in hepatocellular carcinoma. A determinant of chemotherapy response. Am. J. Clin. Pathol..

[12] Kato A, Miyazaki M, Ambiru S, Yoshitomi H, Ito H, Nakagawa K, Shimizu H, Yokosuka O, Nakajima N (2001). Multidrug resistance gene (MDR-1) expression as a useful prognostic factor in patients with human hepatocellular carcinoma after surgical resection. J. Surg. Oncol..

[13] Zhou S, Schuetz JD, Bunting KD, Colapietro AM, Sampath J, Morris JJ, Lagutina I, Grosveld GC, Osawa M, Nakauchi H, Sorrentino BP (2001). The ABC transporter Bcrp1/ABCG2 is expressed in a wide variety of stem cells and is a molecular determinant of the side-population phenotype. Nat. Med..

[14] Zhang G, Wang Z, Luo W, Jiao H, Wu J, Jiang C (2013). Expression of potential cancer stem cell marker ABCG2 is associated with malignant behaviors of hepatocellular carcinoma. Gastroenterol. Res. Pract..

[15] Shen DW, Lu YG, Chin KV, Pastan I, Gottesman MM (1991). Human hepatocellular carcinoma cell lines exhibit multidrug resistance unrelated to MRD1 gene expression. J. Cell Sci..

[16] Cojoc M, Mäbert K, Muders MH, Dubrovska A (2015). A role for cancer stem cells in therapy resistance: Cellular and molecular mechanisms. Semin. Cancer Biol..

[17] Karakasiliotis I, Mavromara P (2015). Hepatocellular carcinoma: From hepatocyte to liver cancer stem cell. Front. Physiol..

[18] Ashokachakkaravarthy K, Pottakkat B (2020). Mitotic quiescence in hepatic cancer stem cells: An incognito mode. Oncol. Rev..

[19] Abdel-Rahman O (2013). Systemic therapy for hepatocellular carcinoma (HCC): From bench to bedside. J. Egypt. Natl. Cancer Inst..

[20] Nio K, Yamashita T, Kaneko S (2017). The evolving concept of liver cancer stem cells. Mol. Cancer.

[21] Cao L, Zhou Y, Zhai B, Liao J, Xu W, Zhang R, Li J, Zhang Y, Chen L, Qian H, Wu M, Yin Z (2011). Sphere-forming cell subpopulations with cancer stem cell properties in human hepatoma cell lines. BMC Gastroenterol..

[22] Chen X, Lingala S, Khoobyari S, Nolta J, Zern MA, Wu J (2011). Epithelial mesenchymal transition and hedgehog signaling activation are associated with chemoresistance and invasion of hepatoma subpopulations. J. Hepatol..

[23] Chen Y, Yu D, Zhang H, He H, Zhang C, Zhao W, Shao RG (2012). CD133(+)EpCAM(+) phenotype possesses more characteristics of tumor initiating cells in hepatocellular carcinoma Huh7 cells. Int. J. Biol. Sci..

[24] Karagonlar ZF, Akbari S, Karabicici M, Sahin E, Avci ST, Ersoy N, Ates KE, Balli T, Karacicek B, Kaplan KN, Celiker C, Atabey N, Erdal E (2020). A novel function for KLF4 in modulating the de-differentiation of EpCAM(-)/CD133(-) nonstem cells into EpCAM(+)/CD133(+) liver cancer stem cells in HCC cell line HuH7. Cells.

[25] Wang Z, Sun M, Li W, Fan L, Zhou Y, Hu Z (2020). A novel CD133- and EpCAM-Targeted liposome with redox-responsive properties capable of synergistically eliminating liver cancer stem cells. Front. Chem..

[26] Kim H, Lee KW, Oh SC, Park MY, Seo S, Jin XL, Hong SK, Yoon KC, Yi NJ, Suh KS (2020). Sirolimus and MMF are insufficient immunosuppressants for regulation of the proliferation of CD133+EpCAM+ cell populations in HCC cell lines. Biomed. Rep..

[27] Karacicek B, Erac Y, Tosun M (2019). Functional consequences of enhanced expression of STIM1 and Orai1 in Huh-7 hepatocellular carcinoma tumor-initiating cells. BMC Cancer.

[28] Thorn CF, Oshiro C, Marsh S, Hernandez-Boussard T, McLeod H, Klein TE, Altman RB (2011). Doxorubicin pathways: Pharmacodynamics and adverse effects. Pharmacogenet. Genomics.

[29] Advani R, Fisher GA, Lum BL, Hausdorff J, Halsey J, Litchman M, Sikic BI (2001). A phase I trial of doxorubicin, paclitaxel, and valspodar (PSC 833), a modulator of multidrug resistance. Clin. Cancer Res..

[30] Mayer U, Wagenaar E, Dorobek B, Beijnen JH, Borst P, Schinkel AH (1997). Full blockade of intestinal P-glycoprotein and extensive inhibition of blood-brain barrier P-glycoprotein by oral treatment of mice with PSC833. J. Clin. Investig..

[31] Henrich CJ, Bokesch HR, Dean M, Bates SE, Robey RW, Goncharova EI, Wilson JA, McMahon JB (2006). A high-throughput cell-based assay for inhibitors of ABCG2 activity. J. Biomol. Screen..

[32] Leier I, Jedlitschky G, Buchholz U, Cole SP, Deeley RG, Keppler D (1994). The MRP gene encodes an ATP-dependent export pump for leukotriene C4 and structurally related conjugates. J. Biol. Chem..

[33] Weidner LD, Zoghbi SS, Lu S, Shukla S, Ambudkar SV, Pike VW, Mulder J, Gottesman MM, Innis RB, Hall MD (2015). The inhibitor Ko143 is not specific for ABCG2. J. Pharmacol. Exp. Ther..

[34] Minotti G, Menna P, Salvatorelli E, Cairo G, Gianni L (2004). Anthracyclines: Molecular advances and pharmacologic developments in antitumor activity and cardiotoxicity. Pharmacol. Rev..

[35] O'Brien CA, Kreso A, Jamieson CH (2010). Cancer stem cells and self-renewal. Clin. Cancer Res..

[36] Rameshwar P, Patel S (2013). Tumorsphere passage for breast cancer stem cells. Protocol Exchange.

[37] Zhang X-L, Jia Q, Lv L, Deng T, Gao J (2015). Tumorspheres derived from HCC cells are enriched with cancer stem cell-like cells and present high chemoresistance dependent on the Akt pathway. Anticancer Agents Med. Chem..

[38] Naujokat C, Steinhart R (2012). Salinomycin as a drug for targeting human cancer stem cells. J. Biomed. Biotechnol..

[39] Hu Y, Smyth GK (2009). ELDA: Extreme limiting dilution analysis for comparing depleted and enriched populations in stem cell and other assays. J. Immunol. Methods.

[40] Terris B, Cavard C, Perret C (2010). EpCAM, a new marker for cancer stem cells in hepatocellular carcinoma. J. Hepatol..

[41] Ma S, Chan KW, Hu L, Lee TK, Wo JY, Ng IO, Zheng BJ, Guan XY (2007). Identification and characterization of tumorigenic liver cancer stem/progenitor cells. Gastroenterology.

[42] Yang ZF, Ho DW, Ng MN, Lau CK, Yu WC, Ngai P, Chu PW, Lam CT, Poon RT, Fan ST (2008). Significance of CD90+ cancer stem cells in human liver cancer. Cancer Cell.

[43] Lee TK, Castilho A, Cheung VC, Tang KH, Ma S, Ng IO (2011). CD24(+) liver tumor-initiating cells drive self-renewal and tumor initiation through STAT3-mediated NANOG regulation. Cell Stem Cell.

[44] Zhu Z, Hao X, Yan M, Yao M, Ge C, Gu J, Li J (2010). Cancer stem/progenitor cells are highly enriched in CD133+CD44+ population in hepatocellular carcinoma. Int. J. Cancer.

[45] Haraguchi N, Ishii H, Mimori K, Tanaka F, Ohkuma M, Kim HM, Akita H, Takiuchi D, Hatano H, Nagano H, Barnard GF, Doki Y, Mori M (2010). CD13 is a therapeutic target in human liver cancer stem cells. J. Clin. Investig..

[46] Xin HW, Ambe CM, Hari DM, Wiegand GW, Miller TC, Chen JQ, Anderson AJ, Ray S, Mullinax JE, Koizumi T, Langan RC, Burka D, Herrmann MA, Goldsmith PK, Stojadinovic A, Rudloff U, Thorgeirsson SS, Avital I (2013). Label-retaining liver cancer cells are relatively resistant to sorafenib. Gut.

[47] Rountree CB, Ding W, He L, Stiles B (2009). Expansion of CD133-expressing liver cancer stem cells in liver-specific phosphatase and tensin homolog deleted on chromosome 10-deleted mice. Stem Cells.

[48] Yin S, Li J, Hu C, Chen X, Yao M, Yan M, Jiang G, Ge C, Xie H, Wan D, Yang S, Zheng S, Gu J (2007). CD133 positive hepatocellular carcinoma cells possess high capacity for tumorigenicity. Int. J. Cancer.

[49] Yamashita T, Ji J, Budhu A, Forgues M, Yang W, Wang HY, Jia H, Ye Q, Qin LX, Wauthier E, Reid LM, Minato H, Honda M, Kaneko S, Tang ZY, Wang XW (2009). EpCAM-positive hepatocellular carcinoma cells are tumor-initiating cells with stem/progenitor cell features. Gastroenterology.

[50] Park DJ, Sung PS, Kim JH, Lee GW, Jang JW, Jung ES, Bae SH, Choi JY, Yoon SK (2020). EpCAM-high liver cancer stem cells resist natural killer cell-mediated cytotoxicity by upregulating CEACAM1. J. Immunother. Cancer.

[51] Li Y, Farmer RW, Yang Y, Martin RC (2016). Epithelial cell adhesion molecule in human hepatocellular carcinoma cell lines: A target of chemoresistence. BMC Cancer.

[52] Firtina Karagonlar Z, Koç D, Şahin E, Avci ST, Yilmaz M, Atabey N, Erdal E (2016). Effect of adipocyte-secreted factors on EpCAM+/CD133+ hepatic stem cell population. Biochem. Biophys. Res. Commun..

[53] Delman M, Avcı ST, Akçok İ, Kanbur T, Erdal E, Çağır A (2019). Antiproliferative activity of (R)-4'-methylklavuzon on hepatocellular carcinoma cells and EpCAM(+)/CD133(+) cancer stem cells via SIRT1 and Exportin-1 (CRM1) inhibition. Eur. J. Med. Chem..

[54] Kaur G, Dufour JM (2012). Cell lines: Valuable tools or useless artifacts. Spermatogenesis.

[55] Wang C, Wang MD, Cheng P, Huang H, Dong W, Zhang WW, Li PP, Lin C, Pan ZY, Wu MC, Zhou WP (2017). Hepatitis B virus X protein promotes the stem-like properties of OV6(+) cancer cells in hepatocellular carcinoma. Cell Death Dis..

[56] Liu DX, Li PP, Guo JP, Li LL, Guo B, Jiao HB, Wu JH, Chen JM (2019). Exosomes derived from HBV-associated liver cancer promote chemoresistance by upregulating chaperone-mediated autophagy. Oncol. Lett..

[57] Zhao X, Guo X, Xing L, Yue W, Yin H, He M, Wang J, Yang J, Chen J (2018). HBV infection potentiates resistance to S-phase arrest-inducing chemotherapeutics by inhibiting CHK2 pathway in diffuse large B-cell lymphoma. Cell Death Dis..

[58] MacNab GM, Alexander JJ, Lecatsas G, Bey EM, Urbanowicz JM (1976). Hepatitis B surface antigen produced by a human hepatoma cell line. Br. J. Cancer.

[59] Nakabayashi H, Taketa K, Yamane T, Miyazaki M, Miyano K, Sato J (1984). Phenotypical stability of a human hepatoma cell line, HuH-7, in long-term culture with chemically defined medium. Gan.

[60] Konno T (1990). Targeting cancer chemotherapeutic agents by use of lipiodol contrast medium. Cancer.

[61] Bayraktar Y, Balkanci F, Kayhan B, Uzunalimoglu B, Gokoz A, Ozisik Y, Gurakar A, Van Thiel DH, Firat D (1996). A comparison of chemoembolization with conventional chemotherapy and symptomatic treatment in cirrhotic patients with hepatocellular carcinoma. Hepatogastroenterology.

[62] Facciorusso A, Bellanti F, Villani R, Salvatore V, Muscatiello N, Piscaglia F, Vendemiale G, Serviddio G (2017). Transarterial chemoembolization vs bland embolization in hepatocellular carcinoma: A meta-analysis of randomized trials. United Eur. Gastroenterol. J..

[63] Pan C, Wang X, Shi K, Zheng Y, Li J, Chen Y, Jin L, Pan Z (2016). MiR-122 reverses the doxorubicin-resistance in hepatocellular carcinoma cells through regulating the tumor metabolism. PLoS ONE.

[64] Shojaie N, Ghaffari SM (2016). Simultaneous analysis of Wnt and NF-κB signaling pathways in doxorubicin sensitive and methotrexate resistant PLC/PRF/5 cells. Cell J..

[65] Lo RC, Leung CO, Chan KK, Ho DW, Wong CM, Lee TK, Ng IO (2018). Cripto-1 contributes to stemness in hepatocellular carcinoma by stabilizing Dishevelled-3 and activating Wnt/β-catenin pathway. Cell Death Differ..

[66] Shimizu S, Takehara T, Hikita H, Kodama T, Tsunematsu H, Miyagi T, Hosui A, Ishida H, Tatsumi T, Kanto T, Hiramatsu N, Fujita N, Yoshimori T, Hayashi N (2012). Inhibition of autophagy potentiates the antitumor effect of the multikinase inhibitor sorafenib in hepatocellular carcinoma. Int. J. Cancer.

[67] Munoz M, Henderson M, Haber M, Norris M (2007). Role of the MRP1/ABCC1 multidrug transporter protein in cancer. IUBMB Life.

[68] Frank NY, Margaryan A, Huang Y, Schatton T, Waaga-Gasser AM, Gasser M, Sayegh MH, Sadee W, Frank MH (2005). ABCB5-mediated doxorubicin transport and chemoresistance in human malignant melanoma. Cancer Res..

[69] Van der Borght S, Komuta M, Libbrecht L, Katoonizadeh A, Aerts R, Dymarkowski S, Verslype C, Nevens F, Roskams T (2008). Expression of multidrug resistance-associated protein 1 in hepatocellular carcinoma is associated with a more aggressive tumour phenotype and may reflect a progenitor cell origin. Liver Int..

[70] Cheung ST, Cheung PF, Cheng CK, Wong NC, Fan ST (2011). Granulin-epithelin precursor and ATP-dependent binding cassette (ABC)B5 regulate liver cancer cell chemoresistance. Gastroenterology.

[71] Zhou Y, Chen E, Tang Y, Mao J, Shen J, Zheng X, Xie S, Zhang S, Wu Y, Liu H, Zhi X, Ma T, Ni H, Chen J, Chai K, Chen W (2019). miR-223 overexpression inhibits doxorubicin-induced autophagy by targeting FOXO3a and reverses chemoresistance in hepatocellular carcinoma cells. Cell Death Dis..

[72] Dong J, Qin Z, Zhang WD, Cheng G, Yehuda AG, Ashby CR, Chen ZS, Cheng XD, Qin JJ (2020). Medicinal chemistry strategies to discover P-glycoprotein inhibitors: An update. Drug Resist. Update.

[73] Toyoda Y, Takada T, Suzuki H (2019). Inhibitors of human ABCG2: From technical background to recent updates with clinical implications. Front. Pharmacol..

[74] Fischer V, Rodríguez-Gascón A, Heitz F, Tynes R, Hauck C, Cohen D, Vickers AE (1998). The multidrug resistance modulator valspodar (PSC 833) is metabolized by human cytochrome P450 3A. Implications for drug-drug interactions and pharmacological activity of the main metabolite. Drug Metab. Dispos..

[75] Falasca M, Linton KJ (2012). Investigational ABC transporter inhibitors. Expert Opin. Investig. Drugs.

[76] Buschauer S, Koch A, Wiggermann P, Müller M, Hellerbrand C (2018). Hepatocellular carcinoma cells surviving doxorubicin treatment exhibit increased migratory potential and resistance to doxorubicin re-treatment in vitro. Oncol. Lett..

[77] Meirelles K, Benedict LA, Dombkowski D, Pepin D, Preffer FI, Teixeira J, Tanwar PS, Young RH, MacLaughlin DT, Donahoe PK, Wei X (2012). Human ovarian cancer stem/progenitor cells are stimulated by doxorubicin but inhibited by Mullerian inhibiting substance. Proc. Natl. Acad. Sci. U.S.A..

[78] Zheng X, Cui D, Xu S, Brabant G, Derwahl M (2010). Doxorubicin fails to eradicate cancer stem cells derived from anaplastic thyroid carcinoma cells: Characterization of resistant cells. Int. J. Oncol..

[79] Bhinge KN, Gupta V, Hosain SB, Satyanarayanajois SD, Meyer SA, Blaylock B, Zhang QJ, Liu YY (2012). The opposite effects of doxorubicin on bone marrow stem cells versus breast cancer stem cells depend on glucosylceramide synthase. Int. J. Biochem. Cell. Biol..

