# Supplementary material for: The inhibition of ABCB1/MDR1 or ABCG2/BCRP enables doxorubicin to eliminate liver cancer stem cells
Source: Sci Rep. 2021 May 24;11:10791. doi: 10.1038/s41598-021-89931-9 (PMC8144399; doi:10.1038/s41598-021-89931-9)

Supplementary Materials

Title: The inhibition of ABCB1/MDR1 or ABCG2/BCRP enables doxorubicin to eliminate liver cancer stem cells

Authors: Wang Yin[1#], Dongxi Xiang[2,3,4#], Tao Wang[5,6], Yumei Zhang[1], Cuong V. Pham[1], Shufeng Zhou[7], Guoqin Jiang[8], Yingchun Hou[9], Yimin Zhu[10], Yinglu Han[11], Liang Qiao[12], Phuong H-L Tran[1*] and Wei Duan[1,*]

Affiliations: [1]Deakin University, School of Medicine, IMPACT, Institute for Innovation in Physical and Mental health and Clinical Translation, Geelong, Victoria, 3216, Australia

[2]State Key Laboratory of Oncogenes and Related Genes, Shanghai, 200127, China

[3]Department of Biliary-Pancreatic Surgery, Renji Hospital Affiliated to Shanghai Jiao Tong University School of Medicine, Shanghai, 200127, China

[4]Shanghai Key Laboratory of Biliary Tract Disease Research, Shanghai, 200092, China

[5]Centre for Comparative Genomics, Murdoch University, Perth, WA 6150, Australia

[6]School of Nursing, Zhengzhou University, Zhengzhou, 450001, China

[7]Department of Chemical Engineering & Pharmaceutical Engineering, College of Chemical Engineering, Huaqiao University, Xiamen 361021, China

[8]Department of General Surgery, Second Affiliated Hospital of Soochow University, 1055 Sanxiang Road, Suzhou 215004, China

[9]Laboratory of Tumor Molecular and Cellular Biology, College of Life Sciences, Shaanxi Normal University, 620 West Chang'an Avenue, Xi'an, Shaanxi 710119, China

[10]CAS Key Laboratory of Nano-Bio Interface, Suzhou Institute of Nano-Tech and Nano-Bionics, Chinese Academy of Sciences, Suzhou 215123, China

[11]Shanghai OneTar Biomedicine, Shanghai 201203, China

[12]Storr Liver Centre, Westmead Institute for Medical Research, University of Sydney and Westmead Hospital, Westmead, NSW 2145, Australia

#These authors contributed equally to this work.

***Corresponding authors**: Phuong H-L Tran. Tel: +61 3 522 73255. Email: phuong.tran1@deakin.edu.au. Wei Duan. Tel: +61 3 522 71149. Email: wei.duan@deakin.edu.au.

Supplementary Methods

Cell lines and cell culture

A549 (human lung carcinoma, ATCC® 185™) cell line was purchased from ATCC. The cells were cultured in DMEM medium supplemented with 10% fetal bovine serum and 1 × Glutamax (Life Technologies, US) in a humidified atmosphere containing 5% CO_2 at 37 °C.

MDR1 function assay using calcein-AM extrusion

The calcein acetoxymethyl ester (calcein-AM) assay was used to determine the activity of MDR1 according to a modified method [1]. Briefly, Huh7 and PLC/PRF/5 cells were seeded in black 96-well plates at a density of 1×10^5 cells per well overnight and treated with 50 µL of verapamil (10, 20 and 50 µM) and valspodar (0.5, 1 and 2 µM) respectively, at 37 °C for 30 min. Then, 50 µL of 2 µM calcein-AM (Sigma) were added into each well. The fluorescence of calcein was immediately measured every 5 min for 1 h using a microplate reader (PerkinElmer, Victor X5) with excitation at 485 nm and emission at 589 nm at room temperature. For pretreatment experiments, cells were incubated for 30 min and washed, and then 50 µL of fresh saline buffer was added before the addition of calcein-AM. The % relative fluorescence (FL) in the cells was expressed as:

% FL = [($FL_{treatment}$ - $FL_{nontreatment}$)/$FL_{nontreatment}$] × 100%.

ABCG2 function assay using Hoechst 33342 extrusion

The Hoechst 33342 accumulation assay is used to investigate the activity of the ABCG2 drug efflux pump [2]. The ABCG2 inhibitor ko143 was dissolved in DMSO. The highest final concentration of DMSO in the cell culture medium for the assays was not more than 0.1%. The Huh7, PLC/PRF/5 and A549 cells were plated into the black 96-well plates respectively at a density of 3×10^4 cells per well and incubated under 5% CO_2 at 37 °C for 24 h. The cell culture media were refreshed the next day with 200 µL phenol-red-free media. Cells were then incubated with various concentrations of ko143 as indicated for 30 min before the addition of 1 µM Hoechst 33342. Fluorescence was measured immediately at constant intervals (60 seconds) for 120 minutes with an excitation of 355 nm and an emission wavelength of 460 nm at 37 °C using a plate reader. The % relative fluorescence (FL) in the cells was expressed as: %FL = [($FL_{treatment}$ - $F_{nontreatment}$)/$FL_{nontreatment}$] × 100%.

siRNA transfection

The dicer-substrate double-strand short interfering RNAs (siRNAs) were derived from previously published 21-mer, which were confirmed to have a high knockdown efficiency [3, 4], and were extended to 27-mer dicer-substrate siRNA for this study to enhance the silencing efficiency [5]. The scrambled siRNAs have the same nucleotide composition as the MDR1 or ABCG2 siRNA but lack significant sequence homology to the human genome. The scrambled siRNAs were designed using the InvivoGen online tool (http://www.invivogen.com/sirnawizard/scrambled.php) and verified using the BLAST software tool (https://blast.ncbi.nlm.nih.gov/Blast.cgi). The siRNAs were synthesized by Integrated DNA Technologies followed by HPLC purification. Supplementary Table ST2 summarizes the sequences of siRNAs and primers used in this study.

Twenty-four hours prior to siRNA transfection, cells were plated in a 6-well cell culture plate at a density of 3 × 10^5 cells/well in 1.5 mL/well DMEM medium without antibiotics. The MDR1 siRNA, ABCG2 siRNA, and their corresponding scrambled siRNAs at a concentration of 20 nM were gently diluted in 250 µL of Opti-MEM reduced serum medium, respectively (ThermoFisher). In parallel, 10 µL of Lipofectamine 2000 was diluted in 250 µL Opti-MEM medium. Following incubation of 5 min at room temperature, the diluted siRNA and Lipofectamine 2000 were mixed gently and incubated for 10 minutes at room temperature, followed by the addition of 500 µL of siRNA-Lipofectamine complexes to each well (containing cells and 1.5 mL full DMEM medium in a well). The plate was mixed gently by rocking back and forth. The cells were incubated in a humidified atmosphere containing 5% CO_2 at 37 °C for 48 h followed by the assessment of expression levels of MDR1, ABCG2 using quantitative RT-PCR and Western analysis.

Cellular uptake of doxorubicin (DOX)

The Huh7 and PLC/PRF/5 cells were seeded at 8 × 10^3 cells per well in an 8-chamber slide (Lab-Tek® II, Nunc, US) for 24 h in preparation for confocal microscopy. The cells were treated with MDR1 inhibitor verapamil (10 µM) or valspodar (1 µM), or ABCG2 inhibitor ko143 (1 µM) at 37 °C for 1 h followed by incubation with DOX (400 nM) for 40 min and visualized using confocal microscopy. Semi-quantification of cellular accumulation of DOX was performed using ImageJ software (National Institutes of Health, US).

To quantify DOX accumulation, the cells were washed with PBS after trypsinization and lysed with 200 µL of cell lysate buffer (50 mM Tris, pH 7.5, 375 mM NaCl, 1 mM EDTA, pH 8.0, 8% glycerol, and protein inhibitor cocktail). Four hundred microliter of acetonitrile was added to each sample to precipitate protein. The samples were vortexed for 30 seconds and centrifuged at 20,000 × *g* for 10 min. The supernatant was injected into the HPLC system (Waters e2695, Milford, US) and

the fluorescence of DOX was determined using a fluorescence detector (Waters 2475, Milford, US). The amount of DOX was derived with the aid of a standard curve.

Tumorsphere formation assay

The tumorsphere assay was conducted according to the previously reported protocol [6]. The cells were seeded in 6-well cell culture plates and treated with 1 µM of MDR1 inhibitor valspodar, 10 µM of MDR1 inhibitor verapamil, or 1 µM of ABCG2 inhibitor ko143, and DOX (200 nM for Huh7 and 100 nM for PLC/PRF/5 cells respectively) for 48 h. Alternatively, the cells were treated with 20 nM siRNA to MDR1 or ABCG2 for 6 h followed by a further 42 h culture in the fresh culture medium. Next, the Huh7 cells or PLC/PRF/5 cells were treated with 200 nM or 100 nM of DOX for 48 h. The Huh7 and PLC/PRF/5 cells were harvested at 80% confluence with trypsin digestion and resuspended as single cells in tumorsphere culture media, which was prepared using the DMEM/F12 serum-free media supplemented with 100 units/mL B27 (Gibco, #10889-038), 5 µg/mL insulin (Sigma, #I9278), 20 ng/mL, epidermal growth factor (R&D Systems, #701-02360), 20 ng/mL basic fibroblast growth factor (R&D Systems, #701-23300) following centrifugation (350 ×*g* for 5 min). Cells were then plated into round-bottom 96-well ultra-low attachment plates (Corning, #CLS3474) at a density of 10, 20, and 50 cells per well respectively for tumorsphere formation at 37 °C. For cells that were previously treated with inhibitor plus DOX, the same reagents were added in the tumorsphere culture media for the rest of the whole culture period. For cells that were transfected with siRNA and then incubated with DOX, the same DOX concentration was used in the tumorsphere culture media for the rest of the whole culture period. The cells were incubated in tumorsphere culture media for 7 days. The formation of tumorspheres was visualized by light microscopy. The frequency of tumorspheres was calculated using the online tool http://bioinf.wehi.edu.au/software/elda/index.html.

Supplementary figures

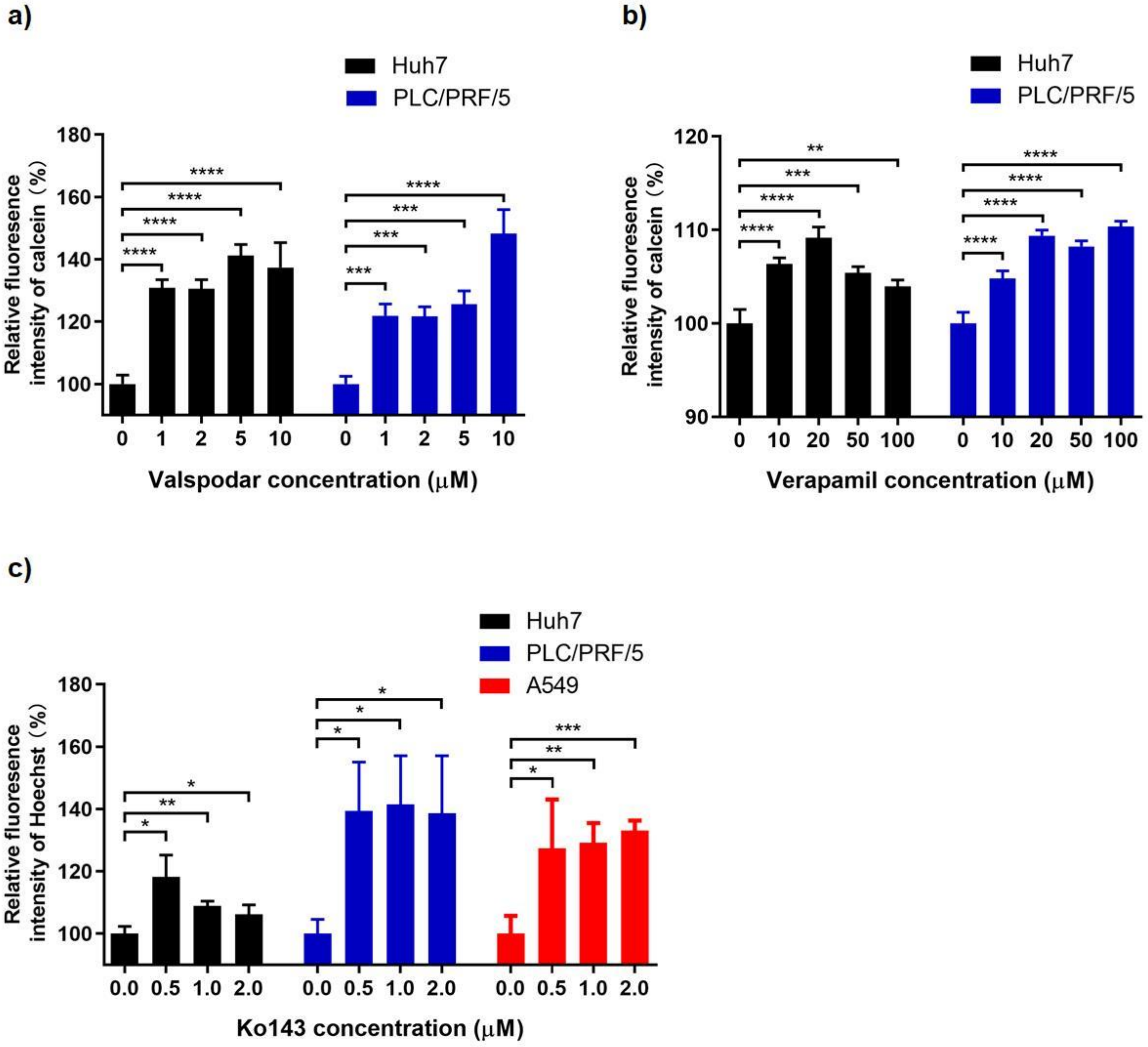

Supplementary Fig. S1. Assessing the drug efflux activity of MDR1 and ABCG2. To measure MDR1 activity, cells were treated with various concentrations of **(a)** valspodar and **(b)** verapamil respectively for 1 h followed by the incubation of calcein-AM for 30 min. The fluorescence intensity of calcein-AM was measured as an indicator of the drug efflux activity. **(c)** To measure ABCG2 efflux activity, cells were treated with a series concentration of ko143 for 30 min, followed by the addition of 10 μM Hoechst 33342 and further incubation for 2 h. The relative intracellular fluorescence intensity of Hoechst was expressed as the ratio of fluorescence intensity of ko143 treated cells to that of untreated cells. Data shown are means ± SD, (n = 3). *** $p<0.001$; **** $p<0.0001$; compared with cells treated with PBS.

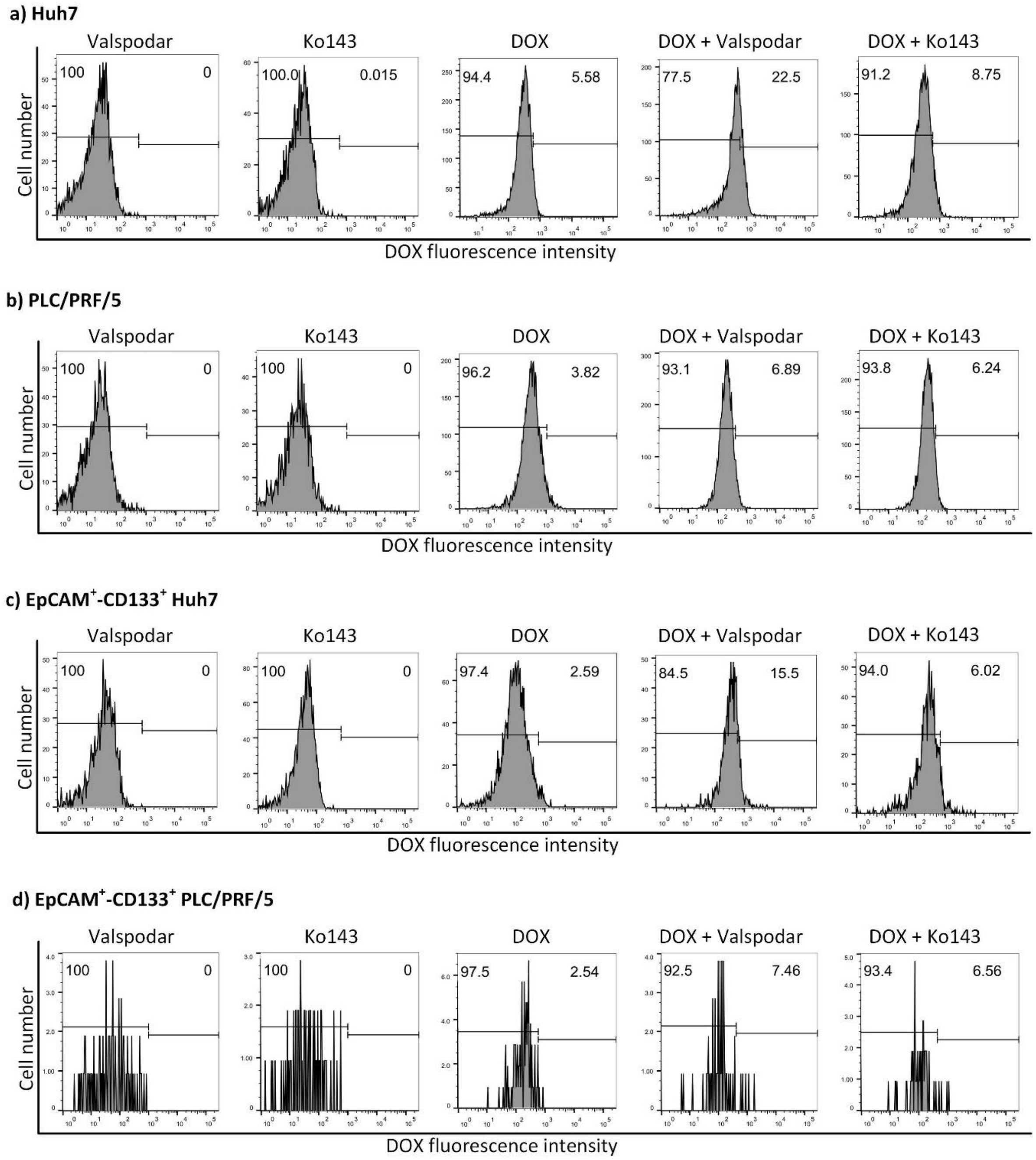

Supplementary Fig. S2. Representative flow cytometric diagrams showing doxorubicin (DOX) accumulation in the bulk population **(a** and **b)** and the EpCAM^{+}-CD133^{+} population **(c and d)** of Huh7 and PLC/PRF/5 cells, respectively. The Huh7 or PLC/PRF/5 cells were treated with 200 nM or 100 nM of DOX, respectively, in the presence or absence of MDR1 inhibitor valspodar (1 µM) or ABCG2 inhibitor ko143 (1 µM) for 24 h followed by being stained with and CD133 antibodies and flow cytometric analysis.

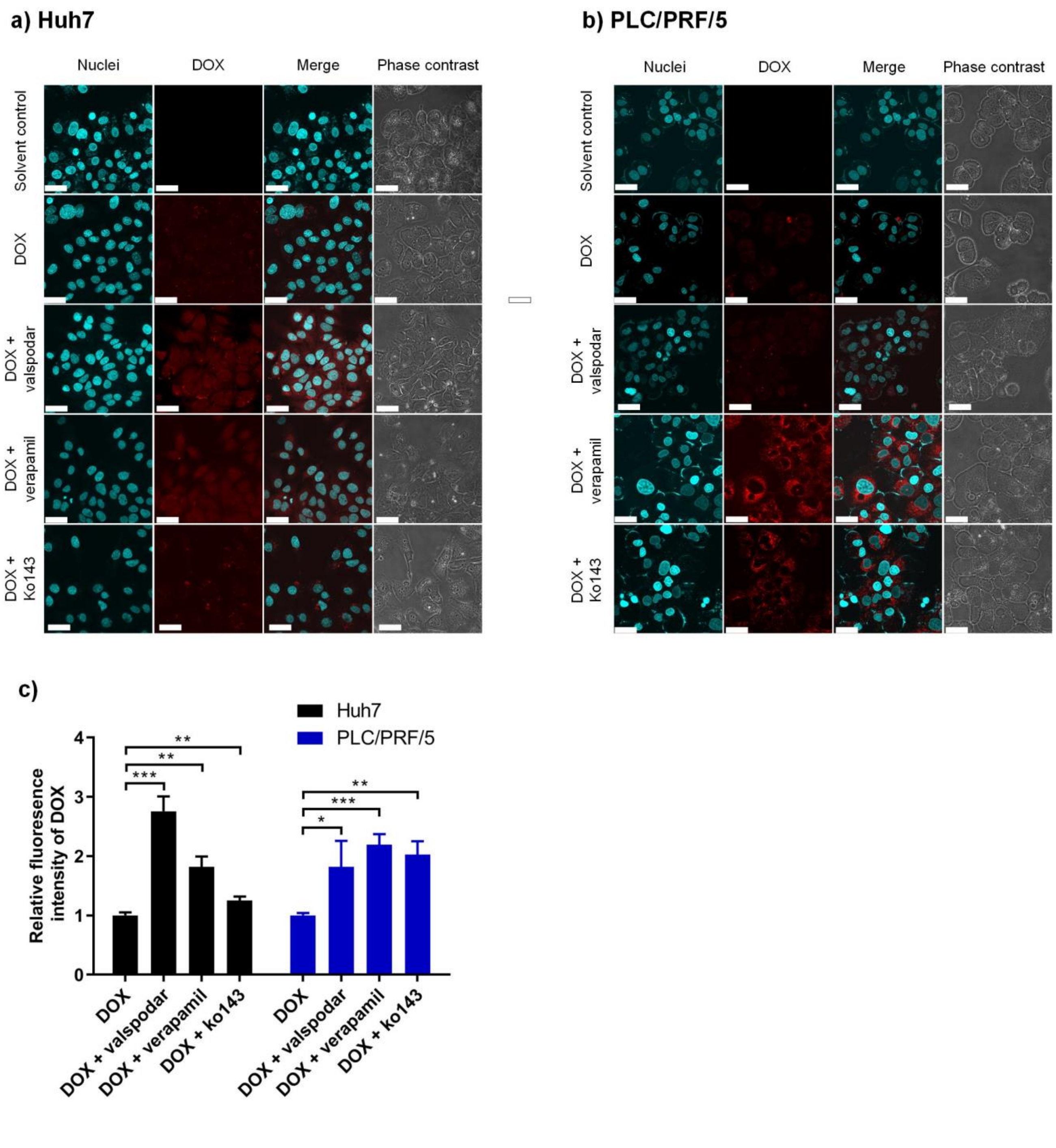

Supplementary Fig. S3. The effect of MDR1 or ABCG2 inhibitor on the intracellular concentration of doxorubicin (DOX). Representative micrographs of Huh7 **(a)** and PLC/PRF/5 **(b)** cells treated with 10 µM verapamil, 1 µM valspodar or 1 µM ko143 at 37 °C for 1 h followed by the incubation of 400 nM DOX for 40 min. **(c)** Quantification of the intracellular DOX concentration after the treatment of valspodar, verapamil, or ko143. Scale bar is 40 µm. *: p<0.05; **: p<0.001; ***: p<0.001, compared with those treated with DOX. Data shown are means ± SD, (n = 3).

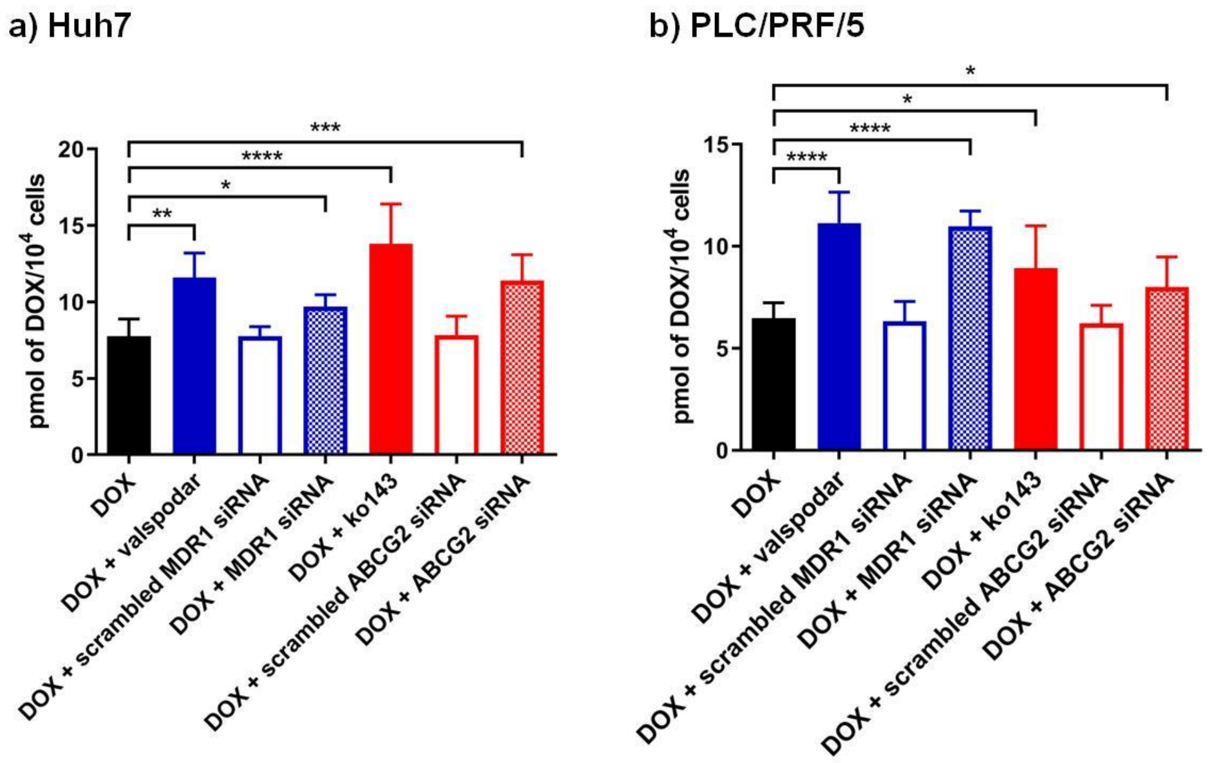

Supplementary Fig. S4. Further quantification of the intracellular doxorubicin (DOX) after the inhibition of MDR1 or ABCG2. The cells were treated with 20 nM siRNA to MDR1 or ABCG2 for 6 h, followed by a further 42 h culture in the fresh culture medium. Alternatively, cells were treated with 1 µM of MDR1 inhibitor valspodar or 1 µM of ABCG2 inhibitor ko143 for 60 min. Cells were then incubated with 100 nM of DOX for 3 h. DOX in the cell lysate of Hun7 **(a)** or PLC/PRF/5 **(b)** were then quantified using HPLC. *: p<0.05; **: p<0.01; ***: p<0.001; ****: p<0.0001 compared with those treated with DOX alone. Data shown are means ± SD, (n = 3).

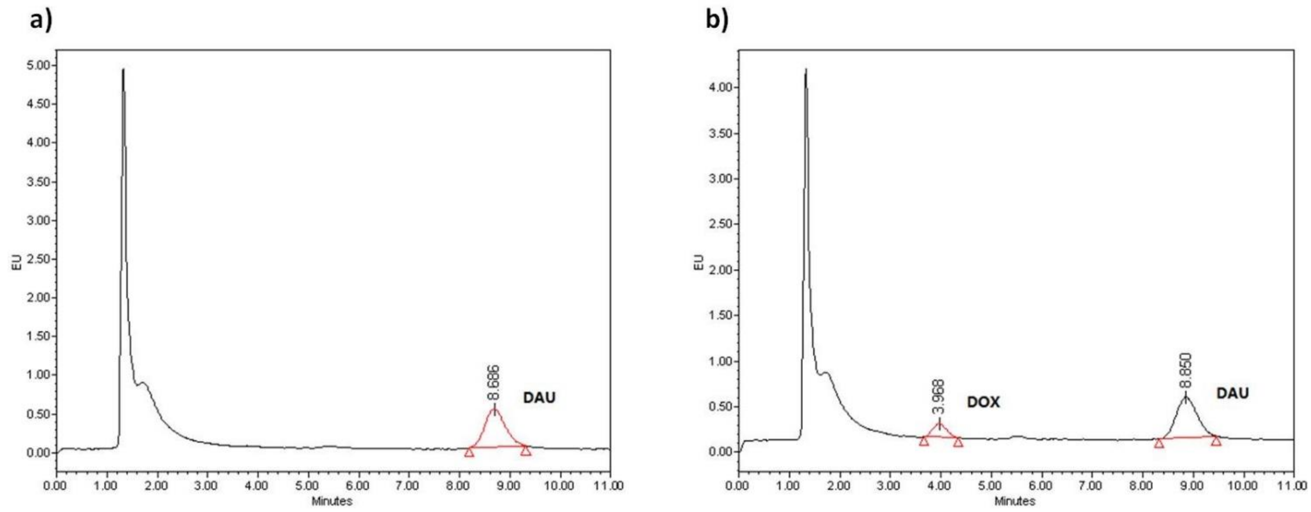

Supplementary Fig. S5. Characterization of the specificity of the HPLC assay for doxorubicin (DOX). Representative chromatograms of blank cell lysate spiked with the internal standard 100 nM daunorubicin (DAU) in the **(a)** absence or **(b)** presence of 10 nM DOX were presented. DOX and DAU were eluted at 4.0 min and 6.7 min, respectively. The endogenous substances showed no interference with the peak of DOX.

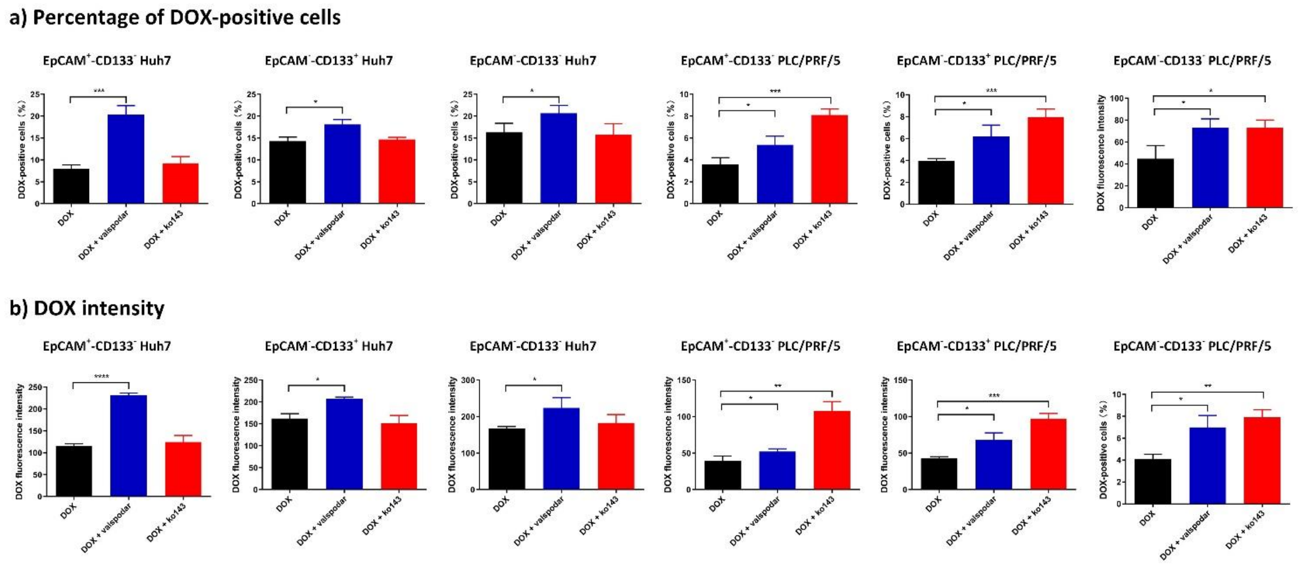

Supplementary Fig. S6. Quantification of intracellular DOX via flow cytometry in the EpCAM$^+$-CD133$^-$, EpCAM$^-$-CD133$^+$, and EpCAM$^-$-CD133$^-$ subpopulations of Huh7 and PLC/PRF/5 cells, respectively. The Huh7 or PLC/PRF/5 cells were treated with 200 nM or 100 nM of DOX, respectively, in the presence or absence of MDR1 inhibitor valspodar (1 µM) or ABCG2 inhibitor ko143 (1 µM) for 24 h followed by flow cytometric analysis. The intracellular retention of DOX measured as **(a)** percentage of DOX-positive cells; **(b)** DOX intensity followed by indicated treatments. Data shown are means ± SD, (n = 3). ****: $p<0.0001$; ***: $p<0.001$; **: $p<0.01$; *: $p<0.05$; compared with DOX-only treatment.

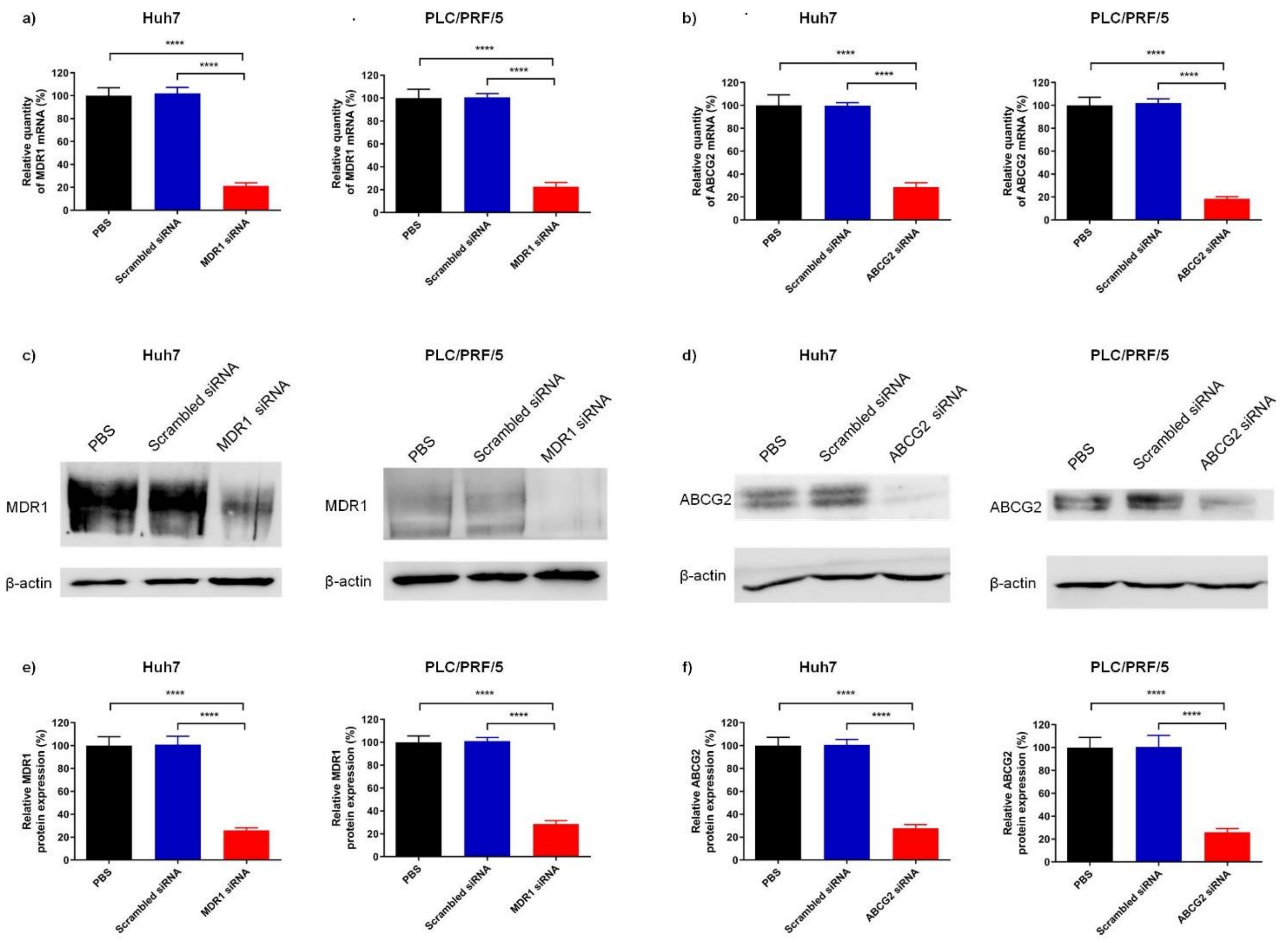

Supplementary Fig. S7. The downregulation of MDR1 and ABCG2 via RNAi. Cells were treated with 20 nM of MDR1 or ABCG2 siRNA for 6 h followed by a further 42 h culture in the fresh culture medium. The mRNA level of MDR1 **(a)** or ABCG2 **(b)** in Huh7 or PLC/PRF/5 cells was determined using RT-qPCR. MDR1 or ABCG2 protein was quantified by Western analysis with β-actin as a loading control. A representative Western blot **(c** and **d)** and the quantification of protein in a given cell line **(e** and **f)** are shown. Data shown are means ± SD, (n = 3). ****: $p<0.0001$ compared with PBS- and scrambled siRNA-treated groups.

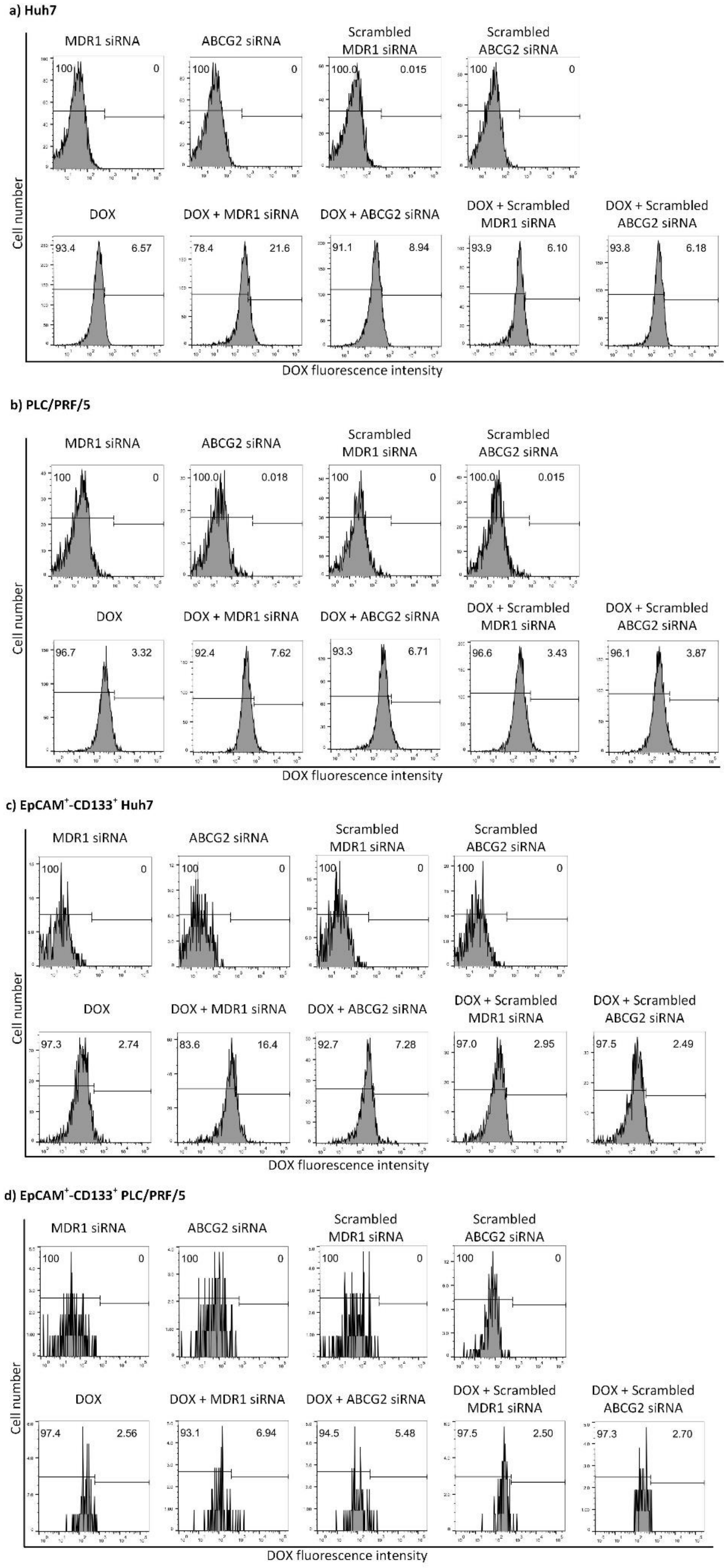

Supplementary Fig. S8. Representative flow cytometric diagrams showing doxorubicin (DOX) accumulation in the bulk population **(a** and **b)** and the EpCAM^{+}-CD133^{+} population **(c** and **d)** of Huh7 and PLC/PRF/5 cells, respectively. The Huh7 and PLC/PRF/5 cells were transfected with MDR1 siRNA (20 nM), ABCG2 siRNA (20 nM) or their corresponding negative control scrambled siRNA (20 nM) for 6 h followed by a further 42 h culture in the fresh culture medium. Huh7 and PLC/PRF/5 cells were then treated with 200 nM or 100 nM of DOX, respectively, for 24 h followed by flow cytometry analysis.

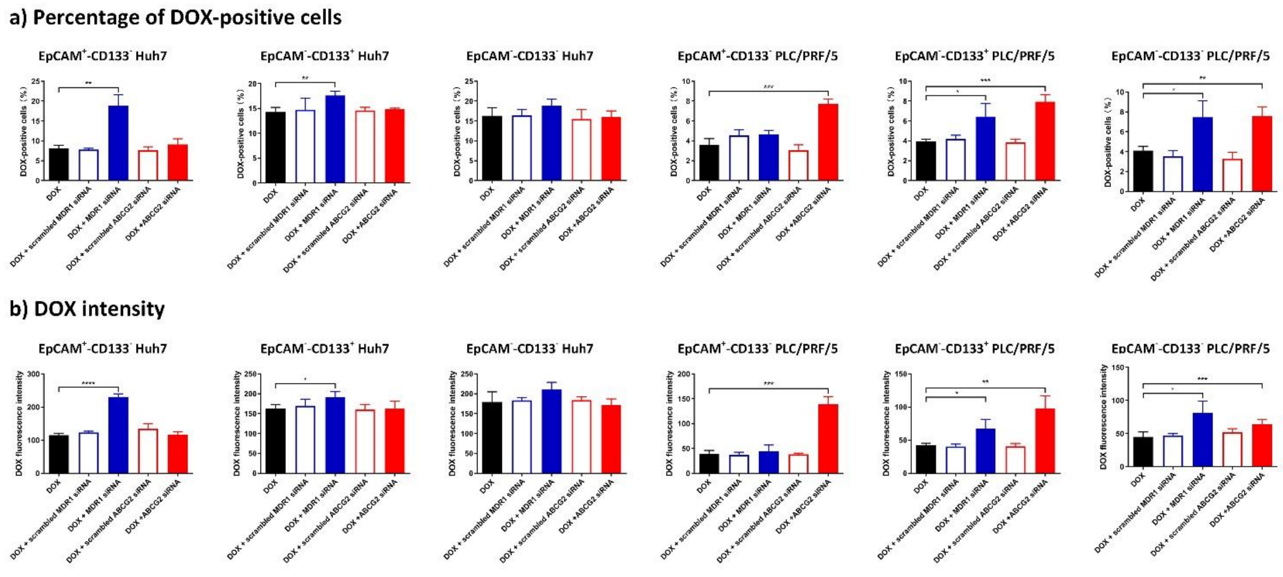

Supplementary Fig. S9. Quantification of intracellular DOX via flow cytometry in the EpCAM$^+$-CD133$^-$, EpCAM$^-$-CD133$^+$, and EpCAM$^-$-CD133$^-$ subpopulations of Huh7 and PLC/PRF/5 cells respectively. Cells were transfected with siRNAs (20 nM) against MDR1 or ABCG2 for 6 h followed by a further 42 h culture in the fresh culture medium. Huh7 and PLC/PRF/5 cells were then treated with 200 nM and 100 nM of DOX, respectively, for 24 h followed by flow cytometry analysis. The intracellular retention of DOX was measured as **(a)** the percentage of DOX-positive cells and **(b)** their DOX fluorescence intensity. Data shown are means ± SD, (n = 3). ****: p<0.0001; ***: p<0.001; **: p<0.01; *: p<0.05; compared with DOX-only treatment.

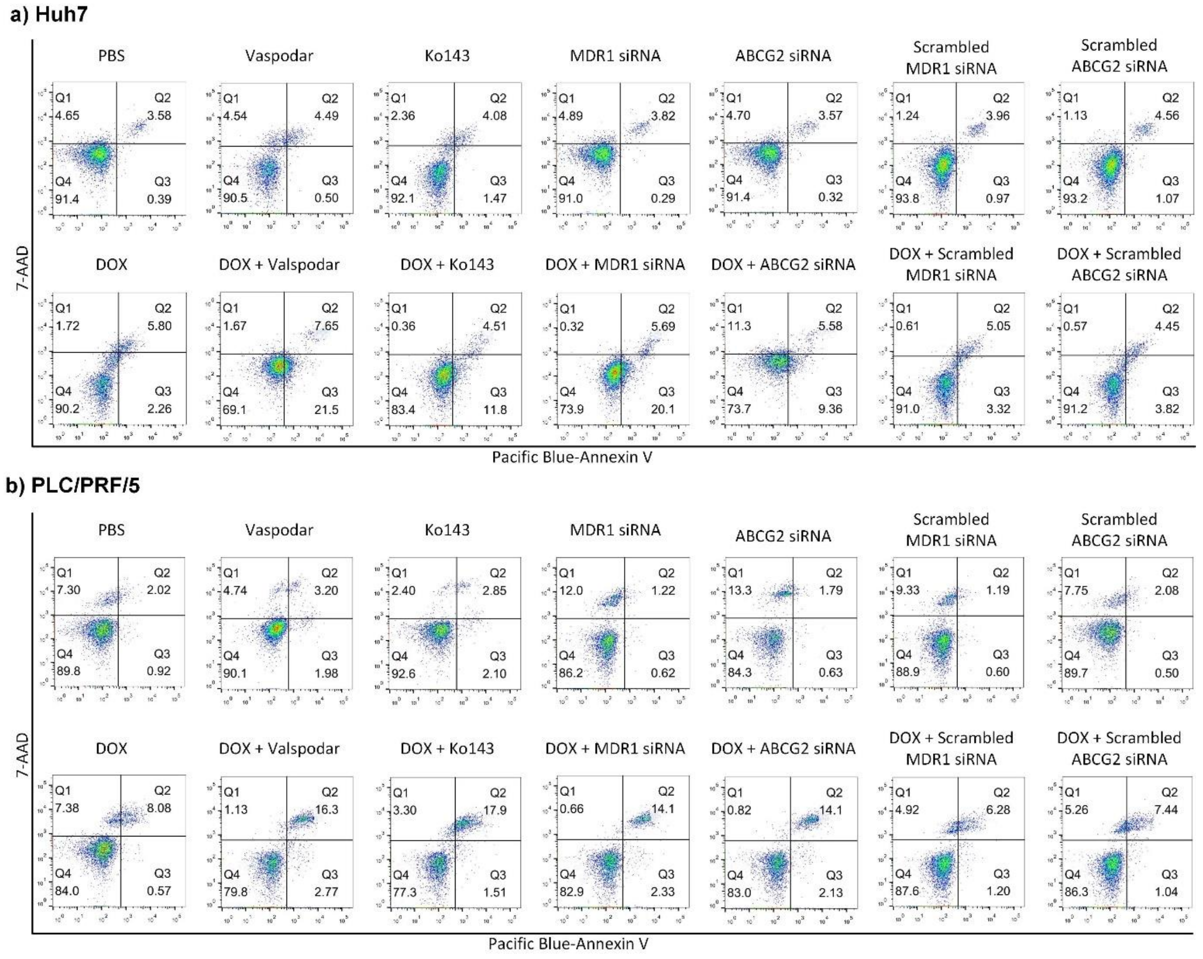

Supplementary Fig. S10. Representative contour diagram of 7-AAD/Annexin V flow cytometry of the bulk population of **(a)** Huh7 and **(b)** PLC/PRF/5 cells. The cells were treated with 1 µM of MDR1 inhibitor valspodar or 1 µM of ABCG2 inhibitor ko143, and DOX (200 nM for Huh7 and 100 nM for PLC/PRF/5 cells, respectively) for 24 h. Alternatively, the cells were treated with 20 nM siRNA to MDR1 or siRNA to ABCG2 for 6 h followed by a further 42 h culture in the fresh culture medium. Next, the Huh7 cells or PLC/PRF/5 cells were treated with 200 nM or 100 nM of DOX for 24 h. The cells were then stained with APC/Fire 750 EpCAM antibody V, VioBright FITC CD133/1 antibody, Annexin V, and 7-AAD for 15 minutes. In each composite panel, the lower left quadrant indicates viable cells (7-AAD$^-$/Annexin V$^-$); the lower right quadrant indicates early apoptotic cells (7-AAD$^-$/Annexin V$^+$); while the upper right quadrant contains late apoptotic cells (7-AAD$^+$/Annexin V$^+$). The combination of early (7-AAD$^-$/Annexin V$^+$) and late (7-AAD$^+$/Annexin V$^+$) apoptotic cells were defined the apoptotic cells.

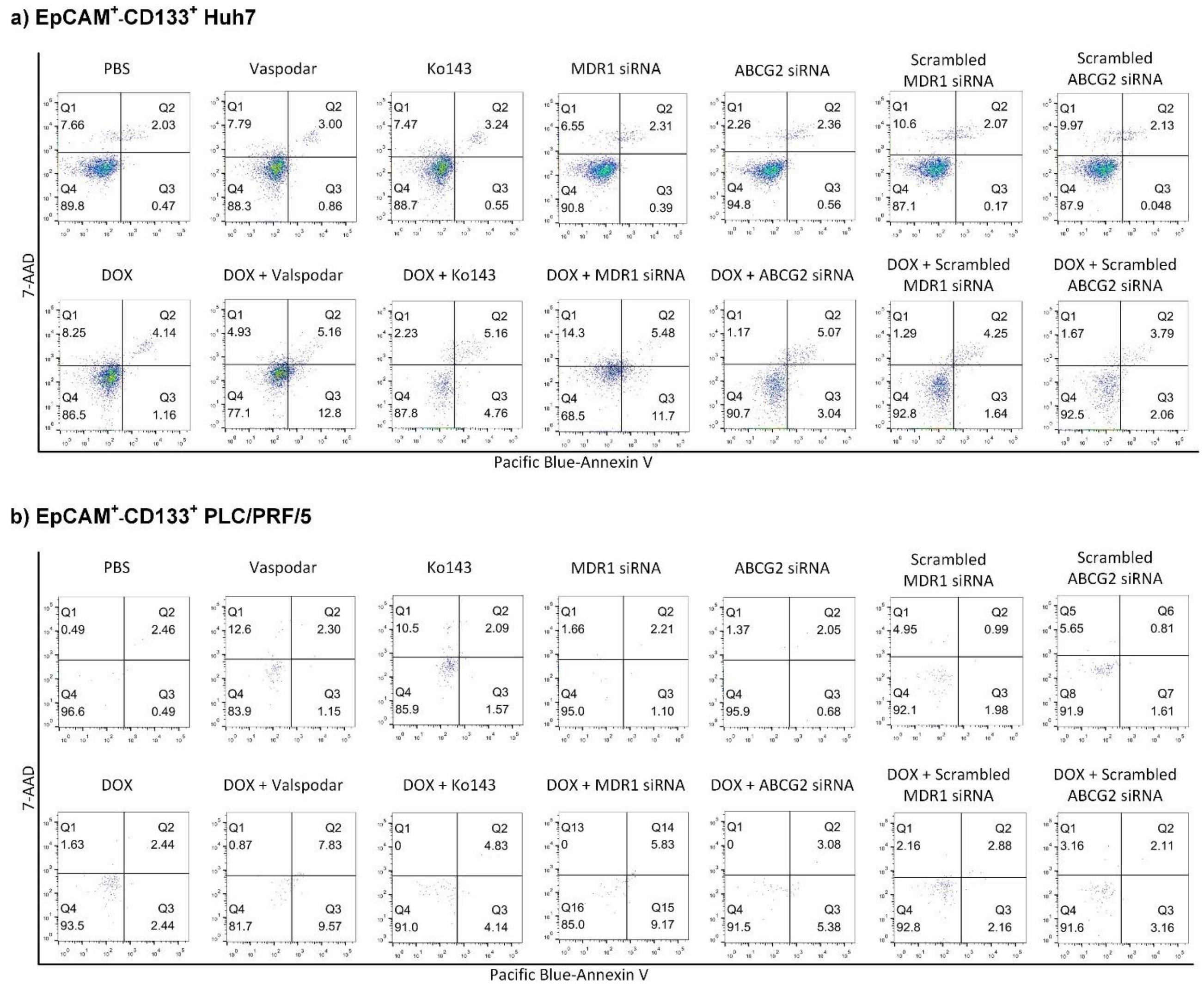

Supplementary Fig. S11. Representative contour diagrams of 7-AAD/Annexin V flow cytometry of the EpCAM$^+$-CD133$^+$ population of **(a)** Huh7 and **(b)** PLC/PRF/5 cells. The cells were treated with 1 µM of MDR1 inhibitor valspodar or 1 µM of ABCG2 inhibitor ko143, and DOX (200 nM for Huh7 and 100 nM for PLC/PRF/5 cells, respectively) for 24 h. Alternatively, the cells were treated with 20 nM siRNA to MDR1 or ABCG2 for 6 hours followed by a further 42 hours culture in the fresh culture medium. Then, the Huh7 cells or PLC/PRF/5 cells were treated with 200 nM or 100 nM of DOX for 24 h. The live or earlier/late apoptotic cells were defined as in the legends of Fig. S10.

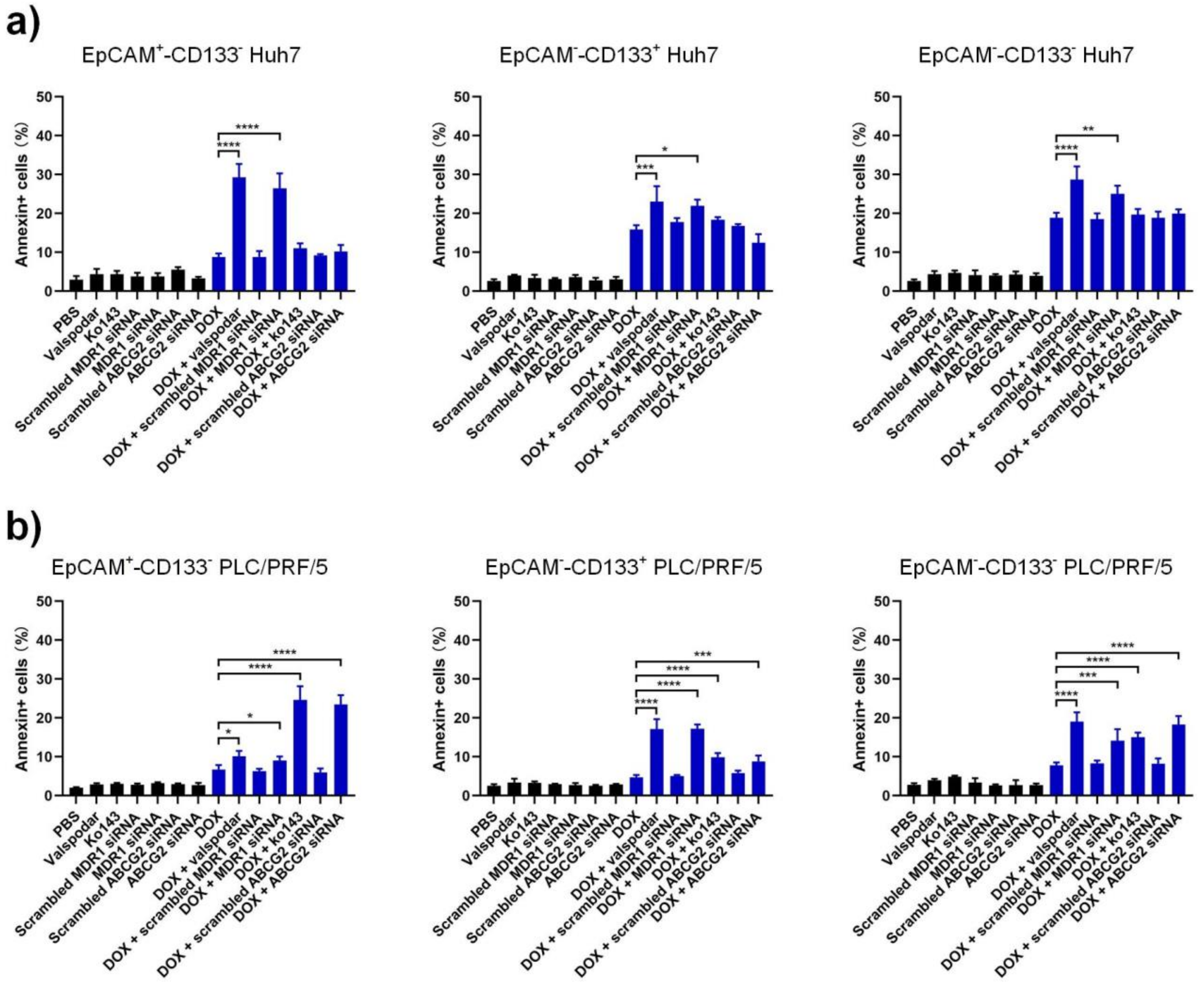

Supplementary Fig. S12. The effect of inhibition of MDR1 and ABCG2 on DOX-induced apoptosis in the EpCAM^{+}-CD133^{-}, EpCAM^{-}-CD133^{+} and EpCAM^{-}-CD133^{+} subpopulations of **(a)** Huh7 and **(b)** PLC/PRF/5 cells respectively. The cells were treated with 1 µM of MDR1 inhibitor valspodar or 1 µM of ABCG2 inhibitor ko143, and DOX (200 nM for Huh7 and 100 nM for PLC/PRF/5 cells, respectively) for 24 h. Alternatively, the cells were treated with 20 nM siRNA to MDR1 or ABCG2 for 6 h, followed by a further 42 hours culture in the fresh culture medium. Then the Huh7 cells or PLC/PRF/5 cells were treated with 200 nM or 100 nM of DOX for 24 h. The percentage of apoptotic cells as defined by the combination of 7-AAD^{-}/Annexin V^{+} and 7-AAD^{+}/Annexin V^{+} cells. Data shown are mean ± SD, n = 3. ****: p<0.0001; *** p<0.001; **: p<0.01; *: p<0.05; compared with DOX-only treatment.

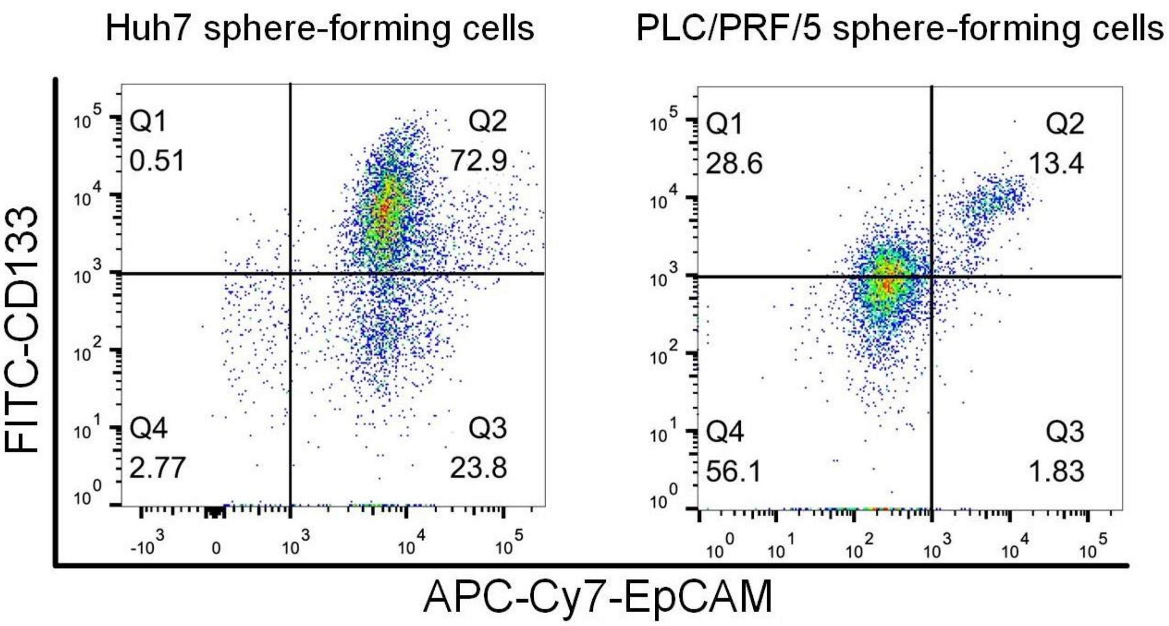

Supplementary Fig. S13. The expression of LCSC markers EpCAM and CD133 in the Huh7 and PLC/PRF/5 sphere-forming cells. The tumorspheres derived from Huh7 or PLC/PRF/5 cells were trypsinized and stained with EpCAM and CD133 antibodies followed by flow cytometry detection.

Supplementary Table ST1. List of antibodies used in the study.

**Antibody**	**Fluorophore**	**Company**	**Cat. No.**
EpCAM	APC/Fire 750	Biolegend	324234
CD133	VioBright FITC	Miltenyl Biotec	130-105-225
MDR1	BV421	BD Biosciences	566015
ABCG2	APC	BD Biosciences	561451
IgG2b, κ isotype control	APC/Fire 750	Biolegend	400372
IgG1, isotype control	VioBright FITC	Miltenyl Biotec	130-104-513
IgG2a, κ isotype control	BV421	BD Biosciences	562439
IgG2b κ isotype control	APC	BD Biosciences	555745

Supplementary Table ST2. Sequences of primers and siRNAs used in this study.

Name	Sequence (5’ – 3’)
MDR1 primer	Sense: 5’-ATATCAGCAGCCCACATCAT-3’<br>Antisense: 5’-GAAGCACTGGGATGTCCGGT-3’
ABCG2 primer	Sense: 5′-TTTCCAAGCGTTCATTCAAAAA-3′<br>Antisense: 5′-TACGACTGTGACAATGATCTGAGC-3′
GAPDH primer	Sense: 5’-GAGCCCCAGCCTTCTCCATG-3’<br>Antisense: 5’-GAAATCCCATCACCATCTTCCAGG-3’
MDR1 siRNA	Sense: 5’-GGAAAAGAAACCAACUGUCAGUGdTdA-3′<br>Antisense: 5′-UACACUGACAGUUGGUUUCUUUUCCUU-3′
ABCG2 siRNA	Sense: 5'-CUGGAGAUGUUCUGAUAAAUGGAdGdC-3′<br>Antisense: 3'-UAGACCUCUACAAGACUAUUUACCUCG-3′
Scrambled MDR1 siRNA	Sense: 5’-AUAAGCGGAAUCAACGAACAAUGdTdA-3′<br>Antisense: 5′-UACAUUGUUCGUUGAUUCCGCUUAUUU-3′
Scrambled ABCG2 siRNA	Sense: 5'-GCAUUGUGAGACGAAUAUUUGGAdGdC-3′<br>Antisense: 3'-UACGUAACACUCUGCUUAUAAACCUCG-3′

Supplementary Table ST3. The tumorsphere incidence induced by doxorubicin (DOX) in Huh7 and PLC/PRF/5 cells after inhibition of MDR1 or ABCG2. Data shown are mean ± SD, n = 3.

Treatment	No. of cells per well	Tumorsphere incidence (out of six)	
		Huh7	PLC/PRF/5
PBS, valspodar, verapamil, ko143, or siRNAs	50	6.00±0.00	6.00±0.00
	20	6.00±0.00	6.00±0.00
	10	6.00±0.00	6.00±0.00
Salinomycin	50	1.00±0.00	0.67±0.58
	20	0.33±0.58	0.33±0.58
	10	0.00±0.00	0.00±0.00
DOX	50	6.00±0.00	6.00±0.00
	20	5.00±0.00	5.33±0.58
	10	5.00±1.00	4.00±1.00
DOX + valspodar	50	2.67±2.08	0.00±0.00
	20	1.00±1.00	0.00±0.00
	10	0.33±0.58	0.00±0.00
DOX + verapamil	50	1.67±1.15	0.00±0.00
	20	1.00±1.00	0.00±0.00
	10	0.33±0.58	0.00±0.00
DOX + scrambled MDR1 siRNA	50	6.00±0.00	6.00±0.00
	20	5.33±0.58	5.33±1.15
	10	3.33±0.58	4.33±0.58
DOX + MDR1 siRNA	50	3.67±0.58	5.67±0.58
	20	2.00±1.00	3.33±0.58
	10	1.00±0.00	2.67±0.58
DOX + ko143	50	6.00±0.00	3.00±1.00
	20	5.00±0.00	2.33±0.58
	10	4.67±0.58	0.67±0.58
DOX + scrambled ABCG2 siRNA	50	6.00±0.00	6.00±0.00
	20	5.33±0.58	5.33±0.58
	10	4.00±1.00	4.33±0.58
DOX + ABCG2 siRNA	50	6.00±0.00	3.33±0.58
	20	5.67±0.58	2.33±0.58
	10	5.67±1.15	1.00±0.00

References

[1] X. Dong, C.A. Mattingly, M.T. Tseng, M.J. Cho, Y. Liu, V.R. Adams, R.J. Mumper, Doxorubicin and paclitaxel-loaded lipid-based nanoparticles overcome multidrug resistance by inhibiting P-glycoprotein and depleting ATP, Cancer Res, 69 (2009) 3918-3926.

[2] M. Galetti, P.G. Petronini, C. Fumarola, D. Cretella, S. La Monica, M. Bonelli, A. Cavazzoni, F. Saccani, C. Caffarra, R. Andreoli, A. Mutti, M. Tiseo, A. Ardizzoni, R.R. Alfieri, Effect of ABCG2/BCRP Expression on Efflux and Uptake of Gefitinib in NSCLC Cell Lines, PLoS One, 10 (2015) e0141795.

[3] H. Wu, W.N. Hait, J.M. Yang, Small interfering RNA-induced suppression of MDR1 (P-glycoprotein) restores sensitivity to multidrug-resistant cancer cells, Cancer Res, 63 (2003) 1515-1519.

[4] M. Bai, M. Shen, Y. Teng, Y. Sun, F. Li, X. Zhang, Y. Xu, Y. Duan, L. Du, Enhanced therapeutic effect of Adriamycin on multidrug resistant breast cancer by the ABCG2-siRNA loaded polymeric nanoparticles assisted with ultrasound, Oncotarget, 6 (2015) 43779-43790.

[5] D.-H. Kim, M.A. Behlke, S.D. Rose, M.-S. Chang, S. Choi, J.J. Rossi, Synthetic dsRNA Dicer substrates enhance RNAi potency and efficacy, Nature Biotechnology, 23 (2005) 222-226.

[6] Y. Hu, G.K. Smyth, ELDA: extreme limiting dilution analysis for comparing depleted and enriched populations in stem cell and other assays, J Immunol Methods, 347 (2009) 70-78.

Original images of Western blots

Supplementary Fig. S7 (c)

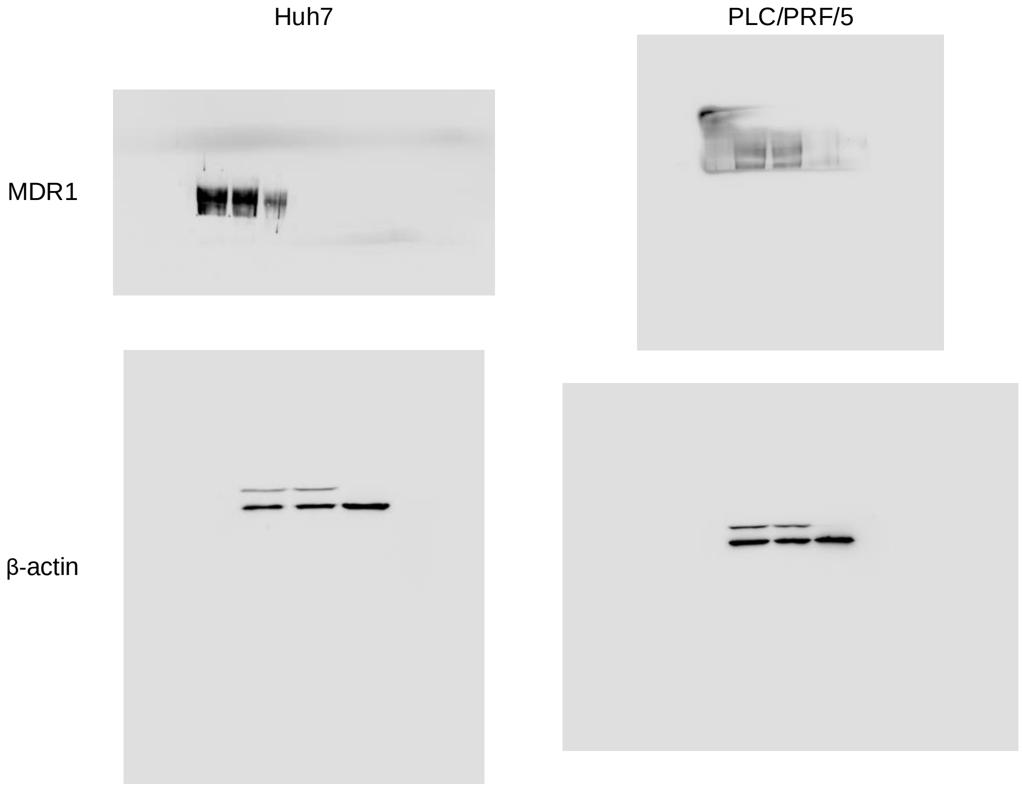

Original images of Western blots (continued)

Supplementary Fig. S7 (d)

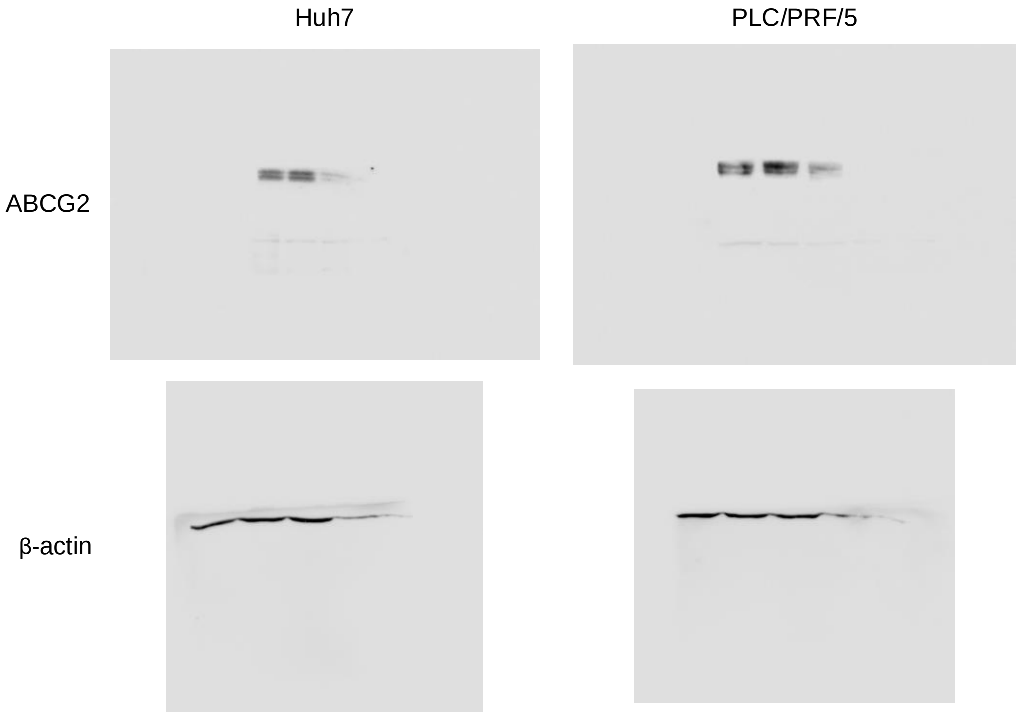

Supplement: Supplementary file 1 — Supplementary Information. [file 41598_2021_89931_MOESM1_ESM.pdf]
